# The Marginal Abatement Cost of Antimicrobials for Dairy Cow Mastitis: A Bioeconomic Optimization Perspective

**DOI:** 10.3390/vetsci10020092

**Published:** 2023-01-25

**Authors:** Ahmed Ferchiou, Youba Ndiaye, Mostafa A. Mandour, Nicolas Herman, Guillaume Lhermie, Didier Raboisson

**Affiliations:** 1 CIRAD, UMR ASTRE, Montpellier, France, ASTRE, CIRAD, INRAE, University Montpellier, Montpellier, Université de Toulouse, ENVT, 31300 Toulouse, France; 2 Department of Animal Wealth Development, Faculty of Veterinary Medicine, Suez Canal University, Ismailia 41522, Egypt; 3 Université de Toulouse, ENVT, 31300 Toulouse, France

**Keywords:** dairy cow, economics, mastitis, antimicrobial use, farmer practices

## Abstract

**Simple Summary:**

The present work proposes an original approach for antimicrobial (AM) resistance reduction based on marginal abatement cost. The optimal use of AMs for dairy cows is defined, and the costs of decreasing antimicrobial use (AMU) below the optimal level and increasing AMU above the optimal level are assessed.

**Abstract:**

Maintaining udder health is the primary indication for antimicrobial use (AMU) in dairy production, and modulating this application is a key factor in decreasing AMU. Defining the optimal AMU and the associated practical rules is challenging since AMU interacts with many parameters. To define the trade-offs between decreased AMU, labor and economic performance, the bioeconomic stochastic simulation model DairyHealthSim (DHS)© was applied to dairy cow mastitis management and coupled to a mean variance optimization model and marginal abatement cost curve (MACC) analysis. The scenarios included three antimicrobial (AM) treatment strategies at dry-off, five types of general barn hygiene practices, five milking practices focused on parlor hygiene levels and three milk withdrawal strategies. The first part of economic results showed similar economic performances for the blanked dry-off strategy and selective strategy but demonstrated the trade-off between AMU reduction and farmers’ workload. The second part of the results demonstrated the optimal value of the animal level of exposure to AM (ALEA). The MACC analysis showed that reducing ALEA below 1.5 was associated with a EUR 10,000 loss per unit of ALEA on average for the farmer. The results call for more integrative farm decision processes and bioeconomic reasoning to prompt efficient public interventions.

## 1. Introduction

Bovine mastitis is an inflammation of the mammary gland characterized by varying degrees of severity, most of which cause loss of milk quantity and quality. Mastitis in dairy cows is a costly disease. A recent meta-analysis assessed the costs of gram-positive and gram-negative clinical mastitis for the farmer to be, on average, EUR 101 and EUR 457 per case, respectively [1]. In industry, mastitis also has a major economic impact by reducing the quality and shelf life of pasteurized milk [2]. Although knowledge and practical rules for managing mastitis in dairy farms have been extensively improved in recent decades, mastitis remains a key issue in the dairy industry since it represents the main reason for antimicrobial use (AMU), which is a hot topic for both industry and society. A recent study showed that during the 2005–2012 period, approximately 60% of AMU in dairy cattle was due to clinical mastitis treatment and preventive treatment at dry-off, with dry-off treatments accounting for 2/3 rds of this 60%. As public health authorities are facing an increasing number of cases of antibiotic-resistant pathogens, many initiatives aim to reduce AMU in animals. In France, the Ecoantibio program implemented since 2012 was associated with an overall 45.4% decrease in AMU through 2020, with AMU in all species considered [3]. The program progress is assessed by using the animal level of exposure to antimicrobials (ALEA) indicator [4]. Despite such efforts, the French dairy sector faces difficulties in decreasing AMU. The specificities of dairy production partly explain the differences in AMU changes observed between species, at least in the French context. The multifactorial origin of mastitis, the long-term production cycle of dairy cows, the economic difficulties faced by dairy farmers in recent decades, the high heterogeneity in farm structures with the associated practices and, finally, the relatively low AMU in the 2010s in cattle compared to other species have all challenged further AMU decreases in the French dairy industry [5,6]. 

Reduced AMU is made possible by advances in mastitis management strategies through optimizing antimicrobial (AM) treatment and disease prevention during lactation and dry-off since [7], but alternatives to AMU should be carefully evaluated to avoid threatening the farm and value chain sustainability [8]. At the farm level, we can identify three potential levers for mastitis-related AMU reduction: optimizing AM treatments in lactation [9], applying selective dry cow therapy (SDCT) [10] and reducing the risk of mammary infections, in particular, by controlling the level of hygiene of the environment in which the animals evolve [11].

Mastitis prevention is based on the control of many risk factors, including drying-off strategies, milking parlor and barn hygiene, feeding, biosecurity, data recording, etc. Commitment to these preventive measures, therefore, requires investments of time and money, which are sometimes difficult to initiate. The difficulty is further increased because economic returns may come later, due to the long production cycle and high value of the animals. Mastitis prevention management and decision-making is, therefore, complicated, due to the multiple associated repercussions that impact the farm dynamics in the long term.

This situation calls for integrative approaches to mastitis management, including the consideration of mastitis economics that include farmer behaviors and daily constraints. Unfortunately, the bioeconomic simulation models commonly reported in the literature are often limited to monetary problem solving and do not consider resource allocation decisions, especially multicriteria decisions, although these decisions are often made by farmers [12].

To our knowledge, no economic analysis has been performed to assess the monetary and non-monetary cost of reducing mastitis-related AMU. The present work aimed to define the trade-offs between mastitis-related AMU decrease, farmer labor and farm economic performance using the bioeconomic stochastic simulation and optimization model DairyHealthSim© (DHS©). The trade-offs were assessed based on the marginal abatement cost (cost in EUR or extra work hours required to decrease AMU by one unit), through the consideration of 2 of the 3 potential levers of AMU reduction, 3 AM treatment strategies at dry-off associated to 5 general barn hygiene practices and 5 milking practices accounting for parlor hygiene levels.

## 2. Materials and Methods

### 2.1. Bioeconomic Modeling

The bioeconomic sequential optimization model DHS© was used. An extensive application of DHS© has been reported previously [12] and a description is reported in Supplemental Data. This model consists of a biological simulation model coupled to an economic optimization model. The biological component aims at the dynamic representation of a dairy herd (Figure S1). Treatment simulation per animal per week allowed us to compute the yearly ALEA at dry-off (ALEA_DO) and for all cows at all production stages as the ratio of treated bodyweight to treatable bodyweight (Equation (1)).

ALEA_DO = Treated bodyweight with AM at dry-off)/(Treatable bodyweight at dry-off)(1)

The recursive mean–variance optimization framework economic model dynamically represented farmers’ input allocation decisions while maximizing utility under constraints [12]. The two constraints used for optimization in the present trial were the workload and AMU [12]. The output of the model is the farmer’s utility and expected income under different combinations of daily activity constraints.

Model calibration details are available in supplemental data for production functions (Tables S1 and S2), feed consumption (Table S3), SCC count simulation (Table S4), diseases functions (Table S1, S6 and S7), treatment functions (Tables S5 and S8), culling management (Table S9), reproduction simulation (Figure S2 and Table S2) and economic parameters (Table S10)

### 2.2. Strategies Tested and Calibration

A deteriorated housing barn and milking parlor hygiene and, therefore, the hygiene of the udder represents a risk factor for intramammary environmental and contagious pathogens [13], while improved cow cleanliness and high milking hygiene would reduce mastitis incidence and improve farm efficiency [14]. SDCT in dairy farms may reduce AMU more than blanket dry-cow therapy (BDCT) [15] and slightly improve farm profitability [16]. However, an improperly implemented SDCT (e.g., not using internal teat sealant) [17] may increase mastitis occurrence and reduce farm profitability [18].

**Figure 1:**
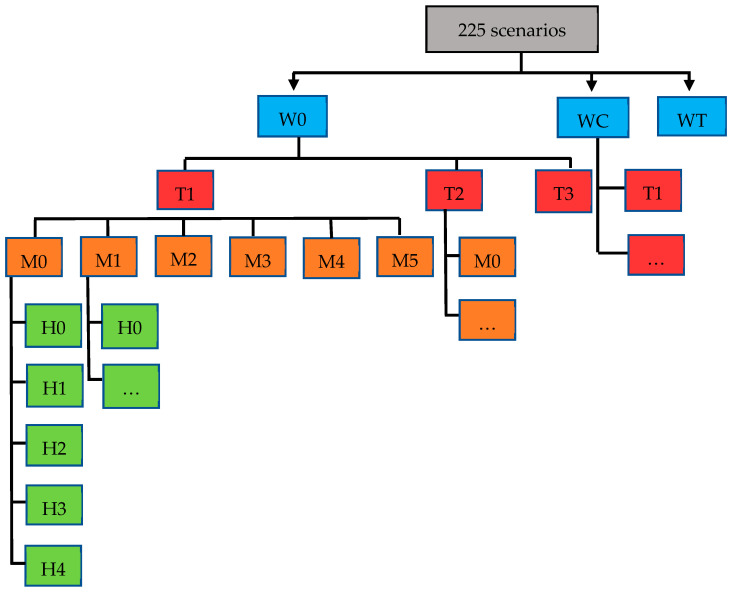
Flowchart describing the scenario combinations. Blue boxes represent milk withdrawal scenarios (W0, WC and WT), red boxes represent treatment at dry-off scenarios (T1, T2 and T3), orange boxes represent milking parlor hygiene practice scenarios (M0, M1, M2, M3 and M4) and green boxes represent dairy housing hygiene practice scenarios (H0, H1, H2, H3 and H4).

Four sets of strategies, including milk withdrawal from the bulk milk tank (W), treatment at dry-off (T), housing barn hygiene (H) and milking parlor hygiene (M), were combined to produce 225 scenarios (Figure 1). 

The set of strategies for treatment at dry-off include: BDCT (T1), improperly implemented SDCT (T2) and a properly implemented SDCT (T3). Sets of strategies for hygiene management represent five levels of farmer practices for both barn and milking parlor hygiene. Three milk withdrawal strategies were implemented to mitigate effects of mastitis incidence in receiving penalties for bulk milk SCC. The sets of strategies for treatment at dry-off (T), housing barn hygiene (H) and milking parlor hygiene (M) influenced mastitis occurrence and, therefore, the AMU and farmer’s workload (Table 1). All sets of strategies influenced economic outcomes. The epidemiologic and economic outcomes of the models were the yearly mean results of 10 years with 50 iterations. The specific parameter values used for scenarios calibration are reported in Table 1 and the base parameters of DHS© are reported in Gables S1.0 to S1.9 in the Part 1 of the Supplemental Data.

### 2.3. Farmer’s ALEA Marginal Abatement Cost

At the farm level, the marginal abatement cost (MAC) of an animal’s AM exposure represents the farmer’s cost of reducing ALEA. The MAC was represented as the income variation due to a change in production strategy for a one-unit reduction in ALEA. The logic of the MAC followed the basic economic theory applied in environmental pollution control, wherein a change in AMU by farmers is efficient when the cost of achieving a specific goal is minimized [22]. The farmer’s ALEA MAC was evaluated by first placing the simulated production inputs (i.e., ALEA or time) and associated incomes in ascending order and then obtaining the dependent variable (Equation (2)) for each milk withdrawal scenario (W):

Income_i_ = f(ALEA_i_, Time_i_, X_i_) (2)

The MAC of ALEA (MAC_ALEA_) was finally defined as the ratio of the difference in income to the difference in ALEA for each scenario (Equation (3)):

MAC_ALEAi_ = (∆Income_i_/∆ALEA_i_) = (Income_i_ − Income_i−1_)/(ALEA_i_ − ALEA_i−1_) (3)

Similarly, the marginal income per additional unit time (was represented as MI_Timei_) follows with Equation (4):

MI_Timei_ = (∆Income_i_/∆Time_i_) = (Income_i+1_ − Income_i_)/(Time_i+1_ − Time_i_) (4)

The graphical representation of MAC_ALEAi_ represents the ALEA marginal abatement cost curve (MACC). The marginal curve facilitated identifying the optimal ALEA situation, which was defined by a marginal cost equal to zero.

## 3. Results

### 3.1. Biological Impacts of Farmers’ Strategies

As expected, the milk withdrawal strategy highly influences the quantity of milk sold (Figure S2.1–S2.3; Supplemental Data). Strategies WT and W0 presented quite similar production levels and income levels, while WC showed deteriorations in the quantity of milk sold and income, particularly in deteriorated hygiene situations. For a given milk withdrawal strategy, the more deteriorated the hygiene (at housing or milking) was, the greater were the reductions in milk production (left to right within strategy T). This trend was the highest for T2. For T2, under deteriorated hygiene conditions, the number of lactating clinical and subclinical mastitis cases increased dramatically as a consequence of a higher risk of contamination during dry-off. Because the milk withdrawal strategy does not impact the milk produced (only the milk sold), the other epidemiologic outcomes were similar regardless of the milk withdrawal strategy (W). ALEA results show that the highest AMU at dry-off was observed under T1, as expected. The highest AMU in all production stages was observed under T2. Moreover, as expected, the lower the housing (H) or milking (M) hygiene was, the higher the AMU in all production stages (left to right in Figure S2.4–S2.6; Supplemental Data). Although both selective treatments at dry-off (T2 and T3) reduced AMU for dried cows, selective treatment with antimicrobials and a teat sealant (T3) provided better mastitis control efficiency per unit of AMU at dry-off. As shown in Supplemental Data Figure S2.7–S2.9, clinical mastitis prevalence followed the same trend as the animals’ level of exposure to antimicrobials. Culling due to low production, subclinical mastitis or recurrent clinical mastitis (the main reasons for culling) was highest under T2 and lowest under T3 (Figure S2.10–S2.12; Supplemental Data).

### 3.2. Bioeconomic Optimization to Define the Best Farmer Strategies

The results highlight that WT_T3 was almost always the optimal strategy (Table 2). When W0 and T3 were not optimal, the difference in risk-adjusted income under the optimal strategy was very limited. The trade-off between the workload and AMU reduction was also demonstrated (Table 2). If the farmer’s objective is to decrease AMU while limiting the workload, housing hygiene must be prioritized over that of the milking parlor (scenarios M2_H1 and M2_H0 produced 10% and 20% AMU decreases, respectively, with limited extra labor). If the workload constraint is not limited, then decreasing AMU is preferentially performed by improving milking hygiene (scenarios M0_H3 and M0_H2 produced 10% and 20% AMU decreases, respectively, with high extra labor). For a strong decrease in AMU, a dramatic increase in workload was required, with more time spent on both barn and milking parlor hygiene. No solution allowed a substantial reduction in AM (more than a 10% reduction) while limiting extra labor within 35 h (grey cells in Table 2).

The changes in revenue between scenarios appeared very significant (Figure S2.1–S2.3; Supplemental Data). Although considering revenues without including workload is restrictive, the results highlight that the highest income was obtained with moderate to good hygiene practices and that the very best hygiene practices were linked to slightly higher revenues compared to those obtained with moderate hygiene practices.

### 3.3. Marginal Abatement Curve Analysis

**Figure 2:**
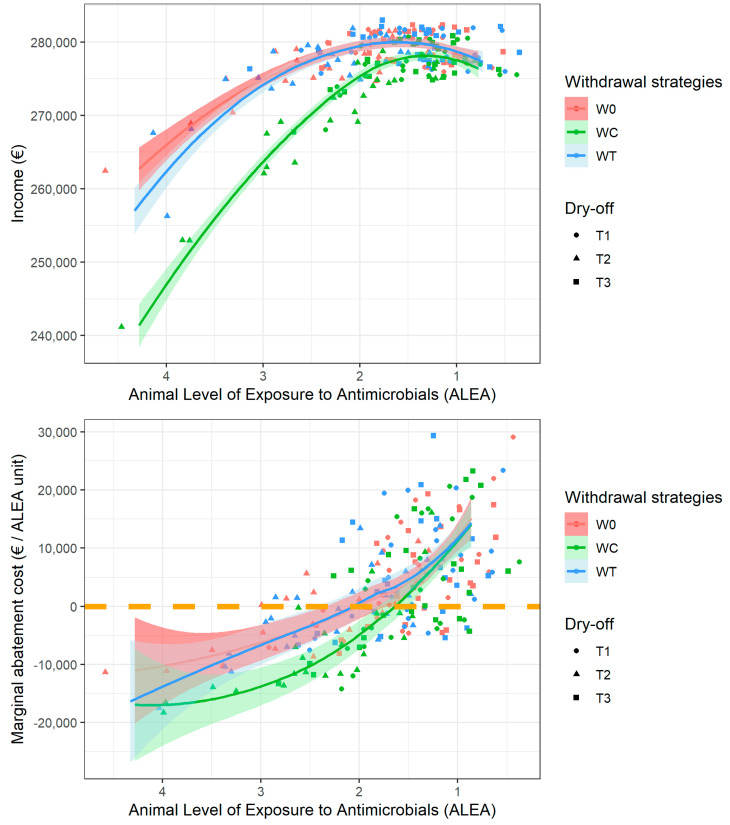
Income (top) and ALEA marginal abatement cost (bottom) according to the ALEA level. The colors represent the milk withdrawal strategies (W0, WC and WT) and the symbols represent the dry-off treatment strategies (T1, T2 and T3).

Considering all the scenarios together facilitated determining the farmer’s average income at each level of ALEA (Figure 2, top) and its marginal evolution (Figure 2, bottom). The average income per unit of ALEA followed an inverted U-shaped curve, with limited income for very high ALEA (left) and a trend of decreasing income for very low ALEA. For W0 and WT (the 2 scenarios of high interest), the income depending on ALEA was very similar, and a peak was observed at ALEA values of 1.75 to 2. The top 10% of incomes were observed for ALEA values of 1–2. The MAC was zero for an ALEA value of two, meaning that the optimal situation is around this value. However, high income variability was observed within this ALEA range. The decreasing relationship between ALEA and MAC (Figure 2, down) was in line with the above figure, which showed an increase in the level of AM exposure associated with a decrease in farmer income. In addition, the farmer MAC curve based on ALEA was almost linear for W0 and WT, and the slope of the line indicated an average of EUR 10,000 in MAC per unit of ALEA reduction. In situations in which the ALEA was less than 2, the ALEA reduction MAC for the farmer was EUR 10,000 per unit, while in situations wherein the ALEA was greater than 2, ALEA reduction generated a gain for the farmer.

**Figure 3:**
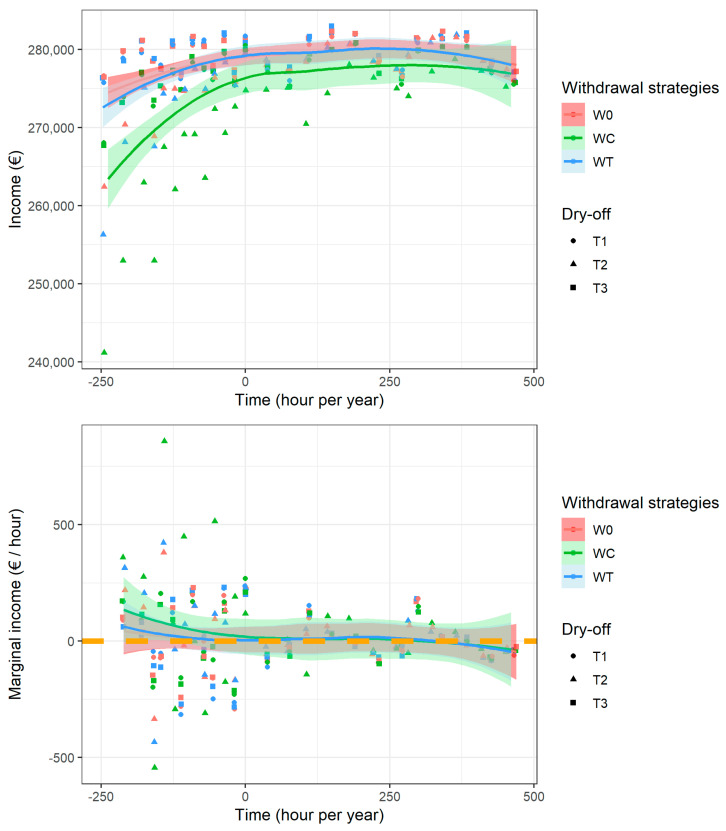
Income (top) and marginal income from time increases (bottom) according to the yearly extra work time required for hygiene management. The colors represent the milk withdrawal strategies (W0, WT and WC), and the symbols represent the dry-off treatment strategies (T1, T2 and T3).

Similarly, the average income per unit of labor time (Figure 3, top) followed an inverted U-shaped curve with a large plateau. The time variable represented the additional farmer workload for milking parlor and barn hygiene maintenance, and a “zero time” situation represented the scenario of average hygiene with no additional risk factors for udder infection. Compared to this average situation, decreasing the average labor time dramatically reduced income, although an increase in labor time only slightly increased income and even reduced income with very high additional labor requirements (>450 extra h of labor per year). This average hygiene situation corresponded to 98% of the maximum income observed in the present simulations. In addition, the marginal income under additional labor time highlights the optimal situation of management strategies close to the average hygiene situation and the associated very low economic motivation to improve hygiene on farms (Figure 3, bottom). For W0 and WT, 1 extra hour of work permitted an extra income of EUR 75/h for farmers with the lowest working time (Figure 3, bottom left). The marginal income per extra hour then decreased quickly to values just above zero (each extra unit time of work was associated with few extra EUR/h income) and, finally, decreased below zero with very high extra working times (each extra hour of work was associated with income loss, as shown in the right side of the figure).

**Figure 4:**
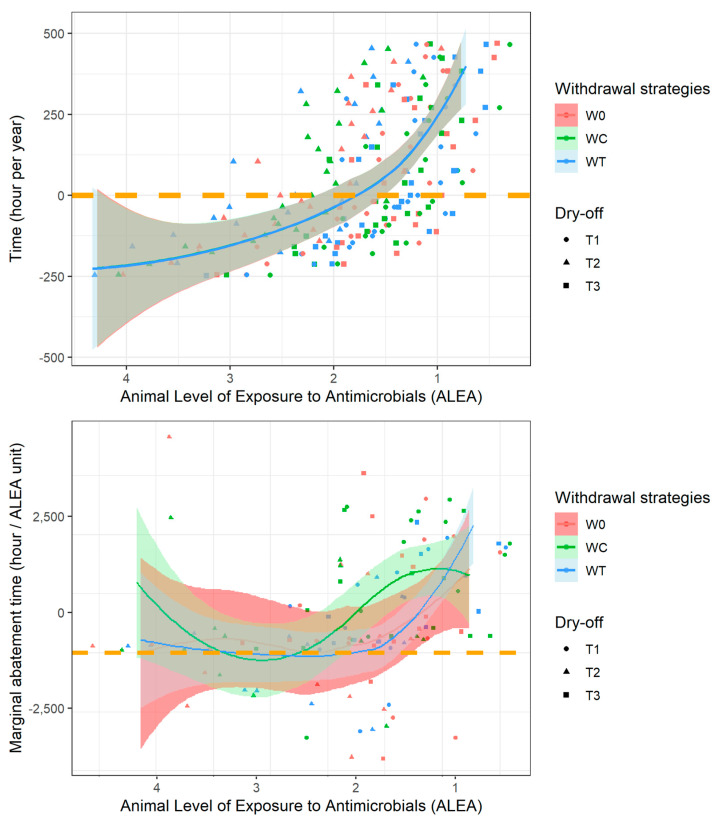
Yearly extra work time required for hygiene management (top) and ALEA marginal abatement time (bottom) according to ALEA. The colors represent the milk withdrawal strategies (W0, WC and WT), and the symbols represent the dry-off treatment strategies (T1, T2 and T3).

As the optimization results showed that no solution made it possible to substantially reduce the ALEA without an additional workload for hygiene tasks, we evaluated the interaction between the additional working time and ALEA (Figure 4, top). A decreasing exponential trend between ALEA and yearly extra work time was observed, demonstrating lesser increases in time required to decrease ALEA for very high to moderate AM users and greater labor increases required for ALEA reductions in other AMU categories, especially for ALEA values below 1.75–2. The substitution relationships between ALEA and time required for hygiene were 343, 210 and 344 h per year for T1, T2 and T3, respectively (T2 < T1, T3; *p* < 0.001). The marginal curve of time required for ALEA variations (Figure 4, bottom) showed no monotonic trend, demonstrating a substitution relationship between time required for barn and milk parlor hygiene. For the WT and W0 strategies, the optimal situations appeared to be ALEA values between 2 and 1.4. Beyond this optimum area, the marginal abatement time greatly increased, demonstrating a high efficiency loss due to time dedicated to farm hygiene.

## 4. Discussion

This study investigates the MAC of AMU by analyzing the trade-offs between mastitis-related AMU reduction, farmer labor and farm economic performance. Many studies identified the effect of management practices for controlling intramammary infections and, therefore, mastitis-related AMU in dairy farms [23,24]. The simulated farm management strategies considered in this work represent different situations of mastitis-related AMU. They concern the implementation of an SDCT in a more or less appropriate way in association with a context of implementation of hygiene measures. The simulation results are consistent with our initial hypotheses which stipulate that the implementation of an SDCT makes it possible to reduce the exposure of animals to antimicrobials. A properly implemented SDCT strategy (T3) demonstrates low epidemiological and economic interest compared to BDCT (T1) [10,16], while an improperly implemented SDCT without teat sealant (T2) is associated with reduced farm economic performance [17]. Regarding hygiene measures, although presenting a challenge to the farmer, particularly in terms of workload, the implementation of cleanliness measures for udder health management can significantly reduce intramammary infections, AMU and the farm’s economic performance [25,26]. Results showed a wide and realistic range of consequences according to hygiene management strategies, including a higher incidence of clinical mastitis infections [11] and a significant preventive cost for the farmer [27], which we assess in monetary and non-monetary terms.

### 4.1. Farmer Decision and MAC

The issue of AMU reduction addresses the multidimensional criteria considered in dairy farmers’ decisions. The animal health perspective has made the leading contribution to economic principles in livestock economics [28]. For a dairy farmer, the main drivers for farm AMU are disease occurrence and the economic and epidemiological benefits of antimicrobial treatments, which are directly related to the farmer’s decision-making regarding the farm’s technical and sanitary management [29,30]. The bioeconomic modeling approach presented in this study evaluated a broad range of farmer management strategies for udder health and treatments at dry-off [12], and a marginal abatement evaluation of AMU was used to evaluate the impacts of AM use reduction on farmer income and resource allocation. The combination of different mastitis management strategies is associated with a wide range of relative risks of mastitis occurrence and, therefore, different levels of AMU. The raw simulation results act as a sensitivity analysis of the risk factor and make it possible to validate the results of the model as to the effect of the management levers for the control of mastitis and AM.

The marginal abatement logic is derived from the basic economic theory of pollution control, suggesting that an allocation of emissions among polluters is efficient if it minimizes the costs of achieving an ambient environmental target. Conceptualized as diffuse pollutants, AMR and GHGs share similar properties that can be explored using the MAC theory of optimal pollution abatement. Although negative externalities of AMU are expected [31], adopting a reduction policy is usually dependent on technical and economic viability if not enforced by legislation. The abatement cost concept explains the costs incurred by firms due to following a new strategy to reduce a problem [32], and some studies, such that of Moran et al. [33], have addressed the farmer’s motivation to adopt new mitigation measures by focusing on profit-maximization behavior. In our research, the abatement cost was conceptualized within the dairy farm context and, more specifically, related to the prevention cost subcategory of the abatement cost. The rationale for using the MAC principle is multifaceted. First, the method facilitated evaluating the impacts of disease control on output loss [34]. Second, this method concept enabled the characterization of the benefits of current AMU by farmers. Third, the MAC principle indicated how reductions can be achieved by demonstrating the cost effectiveness of mitigation measures. Fourth, the abatement cost curve framework offered perspective for setting rational AMU targets. Finally, the MAC theory has considerable explanatory power and has been used in a number of countries (such as the UK, the United States, New Zealand, Ireland and France) to compare a range of agricultural mitigation measures [35].

### 4.2. Empirical Results and Public Policy Perspectives

In the present study, we followed marginal abatement evaluation theory to examine the economic efficiency of antimicrobial use reduction. This study provided a farm-level decision-making tool and yielded interesting insights in terms of public policy implications regarding AMU reduction.

First, this analysis determined the critical point (threshold) of antimicrobial use at which an additional unit of ALEA reduction will not be profitable to the farmer.

The marginal curve of ALEA variation (Figure 2) can be read in both directions for decreased and increased exposure. In suboptimal situations, reducing the ALEA brings additional income to the farmer (negative cost), but an increase in the level of ALEA following the deterioration of hygiene is costly. Both the graphical representation of MAC (Figure 2) and the marginal income of time (Figure 3) show an optimal range of ALEA values from 1.5 to 2 (Figure 4). This optimum is first dependent on the calibration assumption of the model. Model calibration was based on international peer-reviewed publications, and this range is likely to be of interest in many locations, although this assumption needs to be assessed. The identified optimum was also dependent on the limited technical alternatives available for ALEA reduction. While the ALEA MAC followed an almost linear trend, farmer labor costs follow an exponential trend; i.e., with a constant MAC, the working time investment becomes increasingly important for the farmer, which is in line with the model’s calibration parameters.

Second, an additional novelty of this study lies in its focus on finding alternatives to AMU as a production input. The new approach developed in this model allowed the exploration of substitutions between time required for hygiene and ALEA. It also allowed the examination of the time required to reduce ALEA. Farmer workload is a key management constraint and is hardly estimated by farmers. Beyond the cost of mastitis prevention incurred by the farmer workload, the priority need for its evaluation lies in the comparison of allocation among the different possible combinations of management strategies [27].

Working time, which was defined relatively in the biological simulation model, was considered an adjustable variable in economic analyses and not a cost of wages. This innovative approach made it possible to consider the farmer’s working time constraints to refine the optimization results and to observe the marginal income of additional work dedicated to hygiene as well as the time required for AMU reduction.

The results demonstrate that a significant reduction in ALEA can only be achieved with additional time devoted to hygiene, indicating that hygiene is a key factor in reducing the use of antibiotics in dairy farms. The results also show a lower time requirement for hygiene to reduce ALEA under the technical scenario of treatment at dry-off, with which the most mammary infections occurred (220 h for T2). Moreover, the times invested in the barn and milking parlor can be substituted. If the objective is to decrease AMU while limiting the workload, barn hygiene must be prioritized over that of the milking parlor. If the workload is not limited, then decreasing AMU is preferentially achieved through the improvement of milking hygiene. Moreover, in situations wherein the ALEA is lower than the optimum, the time investment required for hygiene is inefficient, but saving time will have little impact on income and the ALEA. The economic value of the farmer’s working time is difficult, if not impossible, to monetize within a framework of limited strategy choices. This analysis should be reproduced in a framework wherein trade-offs of farmer’s working time investment can be evaluated between different dairy farming tasks such as reproduction management, lameness detection and prevention and early detection of other major production diseases. In a sense, the results illustrate a loss of farmer profit if he or she increases his or her level of ALEA, but also the possibility of reducing animal exposure to antibiotics through changes in practice. Even if costs related to hygiene consumables and straw purchases are necessary to improve hygiene and, therefore, reduce the ALEA, the time cost to the farmer remains the main driver of improvement in our context, in which proposals for change are limited to improving farm hygiene.

Third, the optimal ALEA range identified was between 1.5 and 2, and within this optimal zone, an objective that can be achieved by public authorities at a lower cost can be identified. Beyond this level, the use of antibiotics and the time dedicated to farm hygiene are not efficient from the producer’s point of view, and public intervention is necessary. A restrictive public intervention could lead to significant distortion in the dairy value chain [36], and even in countries with low usage, motivation and spillover effects in farmer’s networks seem to be efficient technical levers for reducing the use of antibiotics [37]. For producers, we believe that incentive interventions, such as technical levers [38] and awareness promotion [39] provided at the community level [40], are more effective in efforts to reduce the exposure of production animals to antibiotics, hence the interest in place-based mesoeconomic approaches to support public interventions [41]. The results obtained in this work demonstrate that public interventions must also be targeted to achieve results. An incentive that encourages farmers to devote more time to or facilitates access to agricultural labor for hygiene maintenance will be effective, especially for farmers whose ALEA level is above the optimal range. For farmers whose ALEA level is lower than the optimal level, an incentive measure that provides possible substitutions for farmers’ working time, such as robotization for hygiene maintenance, will be more efficient.

## 5. Conclusions

The present work is one of the rare publications analyzing AMU with an approach based on the MAC. The optimal AMU determined in the present study corresponds to an ALEA of 1.5 to 2, considering both income and labor. We estimated an MAC of EUR 10,000 per unit ALEA for ALEA values below 1.5. The method used in this work is based on bioeconomic modeling associated with a marginal analysis of AMU reduction which focuses on considering mastitis management strategies as alternatives to AMU. AMU and the farmer’s workload are considered as production inputs which allowed us to define the trade-offs between mastitis-related AMU decrease, farmer labor and farm economic performance. Results determined a critical point of AMU beyond which an additional unit of ALEA reduction will not be profitable to the farmer and public intervention is necessary. Above this point, an objective that can be achieved by public authorities at a lower cost can be identified.

## Figures and Tables

**Table 1:** Descriptions and simulation model impact of milk withdrawal (W), treatment at dry-off (T), housing hygiene (H) and milking parlor hygiene (M) scenarios.

	Description	Declination as Impact
**W (milk withdrawal)**		
W0: no milk withdrawal	A cow’s milk is removed from the milk tank when it contains more than 10,000,000 SCC/mL of milk.	
WC: strict cow threshold (SCC) milk withdrawal strategy	A cow’s milk is removed from the milk tank when it contains more than 800,000 SCC/mL of milk.	
WT: mixed cow and tank threshold (SCC) milk withdrawal strategy	A cow’s milk is removed from the milk tank when it contains more than 800,000 SCC/mL of milk only if the milk tank is at more than 300,000 SCC/mL of milk.	
**T (treatment at dry-off)**		
T1: common practice	Systematic antibiotic treatment at dry-off for all cows.	[19]
T2: simple selective antibiotic treatment	Selective antibiotic treatment at dry-off for cows that produced > 250,000 SCC/mL of milk in the previous month.	Relative risk = 2 for clinical mastitis up to 14 WIM in untreated cows (<250,000 SCC/mL of milk) compared to conventional treatment [20].
T3: combined selective antibiotic treatment	Selective antibiotic treatment at dry-off of cows that produced > 250,000 SCC/mL of milk in the previous month and teat sealant for other cows.	Relative risk = 1 for cows treated with antibiotics at dry-off and for cows that received a teat sealant [21].
**H (housing hygiene)**		
H0	Very good dairy housing hygiene (more straw for cows lactating and undergoing dry-off) and higher farmer time investment ^2^.	For lactating cows: 4–6 kg straw/cow/d + 12 s/cow; for cows undergoing dry-off: 5 kg straw/cow/d; relative risk ^1^ of clinical mastitis = 0.7.
H1	Good hygienic measures (relatively more straw for lactating cows and more straw for cows undergoing dry-off) and lower farmer time investment ^2^.	For lactating cows: 3–5 kg straw/cow/d + 6 s/cow; for cows undergoing dry-off: 5 kg straw/cow/d; relative risk ^1^ of clinical mastitis = 0.8.
H2	Average hygienic measures (lesser amount of straw) and the recommended farmer time investment.	For lactating cows: 2–3 kg straw/cow/d + recommended time; for dry-off cows: 3 kg straw/cow/d; relative risk ^1^ of clinical mastitis = 1.
H3	Deteriorated hygienic measures (small amount of straw) and some farmer time savings ^2^.	Lactating: 1.5–3 kg straw/cow/d − 6 s/cow; dry-off: 1.5 kg straw/cow/d; relative risk ^1^ of clinical mastitis = 1.25.
H4	Very deteriorated hygienic measures (small amount of straw) and increased farmer time savings ^2^.	Lactating: 1.5–3 kg straw/cow/d − 12 s/cow; 1.5 kg straw/cow/d; relative risk ^1^ of clinical mastitis = 1.5.
**M (Milking parlor hygiene)**		
M0	Very good hygienic measures, extra farmer time investment and increased consumable use ^3^.	1 min/cow/d + EUR 0.0452; relative risk ^1^ of clinical mastitis = 0.7.
M1	Good hygienic measures, extra farmer time investment and increased consumable use ^3^.	30 s/cow/d + EUR 0.0226; relative risk ^1^ of clinical mastitis = 0.8.
M2	Average hygienic measures, only the recommended farmer time investment and no increased consumable use ^3^.	Time and cost according to recommendations; relative risk ^1^ of clinical mastitis = 1.
M3	Deteriorated hygienic measures, farmer time savings ^2^ and no increased consumable use ^3^.	−7 s/cow/d + EUR 0; relative risk ^1^ of clinical mastitis = 1.25.
M4	Very deteriorated hygienic measures, increased farmer time savings ^2^ and no increased consumable use ^3^.	−15 s/cow/d + EUR 0; relative risk ^1^ of clinical mastitis = 1.5.

Notes: ^1^ Relative risk assumptions were based on the authors’ expertise. ^2^ The additional time corresponds to additional time required relative to average practices, and time savings are the time saved relative to average practices. ^3^ Consumables included udder sanitizers, disinfectants, towels (drying towels and paper towels) and gloves.

**Table 2:** Scenarios of optimal utility according to time and animals’ level of exposure to antimicrobials (ALEA) reduction constraints.

		ALEA reduction constraints				
		0%	10%	20%	30%	40%
**Maximum extra time** **constraint.**	5 h/month	W0_T1_M2_H2	WT_T3_M2_H1			
	10 h/month			WT_T3_M2_H0		
	15 h/month	WT_T3_M1_H3				
	20 h/month			WT_T3_M1_H1		
	25 h/month					
	30 h/month		W0_T3_M0_H3			
	35 h/month			WT_T3_M0_H2	WT_T3_M1_H1	
	40 h/month					WT_T3_M0_H0
	Unlimited					

## Data Availability

Not applicable.

## Supplementary Materials

The following supporting information can be downloaded at: https://www.mdpi.com/article/10.3390/vetsci10020092/s1. Table S1. Biological model input parameters. Table S2. Production functions calibration parameters. Table S3. UFL, crude protein and crude fiber food requirements calculation table. Table S4. Somatic cells count after clinical mastitis infection per pathogen. Table S5. Probability of treatment delay of clinical mastitis infections per pathogen. Table S6. Calibration parameters for calves’ diseases simulation. Table S7. Cows diseases calendar, cross diseases risk calibration and treatment intervention. Table S8. Characteristics of treatments used in DHS. Table S9. Culling decision-making calibration. Table S10. Economic model calibration. Figure S1. DHS biological model overview. Figure S2. Reproduction simulation overview. References [41,42,43,44,45,46,47,48,49,50,51,52,53,54,55,56,57,58,59,60,61,62,63,64,65,66,67,68,69,70,71,72,73,74,75,76,77,78,79,80,81,82] are cited in the supplementary materials.
