# Supplementary material for: The Marginal Abatement Cost of Antimicrobials for Dairy Cow Mastitis: A Bioeconomic Optimization Perspective
Source: Vet Sci. 2023 Jan 25;10(2):92. doi: 10.3390/vetsci10020092 (PMC9962292; doi:10.3390/vetsci10020092)
Supplement: Supplementary file 1 [file vetsci-10-00092-s001.zip › vetsci-2147427-supplementary.pdf]

PART 1: DESCRIPTION OF THE BIOLOGIC MODEL

DairyHealthSim is composed by a biologic model coupled to and economic optimisation. The biologic model is defined on a cow-week basis and weekly probabilities of events, productions and diseases. This biological component aims at the dynamic representation of a dairy herd. From birth to death, each animal was characterized weekly according to its physiological and production statuses (e.g., male calf, female calf, pregnant, lactating cow, and dry cow). The mechanistic model was built so as to avoid a priory within the model: each cow-event was systematically defined – directly or indirectly- through the model (no catch all function such as "all other mortality is gathered in this parameter"). This framework was applied to the 3 main types of functions that are i) production (growth and milk yields, reproduction), ii) diseases (as a damage of production) and iii) treatments and farmer management (as one type of control of damage) (Figure S0). An overview of bibliographic sources for diseases risks and effects; lactation parameters, growth and food needs, reproduction parameters and culling rules calibration parameters is presented in Table S0. When appropriate, others references are provided for each Table.

1- Production functions

1.1. Growth

Animal growth is simulated to estimate calves and cow's body weight from insertion (birth) to herd exit. A calf is born with an initial weight BW_{Birth} and is then characterized by his average daily gain (ADG). The cows live weight is based on Equation 1 [1]:

$$BW_{a,\ wim,\ p\ t} = BW_{Mat}\ [1 - \{1 - (BW_{Birth}\ \ BW_{Mat}\ -1)^{\frac{1}{3}}\}\ \exp(-k\ d)]^3 + p_1\ 7{*}wim\ p_2^{-1} \exp\ (1 - 7\ wim\ p_2^{-1}) + p_3^3\ \ t_{pc}^3\ [1]$$

Where $BW_{a,wim,p\ t}$ stands for body weight of a cow with d days old at week in milk wim and days in pregnancy pt. BW_{MAT} indicates the weight at maturity, LWBirth denotes the birth weight, k denotes the growth rate, and p_1, p_2 and p_3 denote the maximum decrease of live weight during the lactation, the time during the lactation with the minimum live weight and a pregnancy parameter, respectively (Table S1).

During simulation, every animal created is randomly assigned a BW_{MAT} and its body weight is computed every week. This allows weekly estimation of food needs, heifer's reproduction status date and meat production at culling by applying a carcass yield $Carc_{Cull}$ (Table S1)

1.2. Food requirements

Cow's nutritional requirements are computed in the model. This nutritional need is calculated weekly according to the physiological stage of the cow and the health events. Food requirements are expressed in terms of dry matter intake (DMI), feed unit milk (UFL, French equivalent of Net Metabolisable Energy) and crude proteins (CP). The composition of the diet is optimized, on a yearly base, in the economic decision model.

The model first estimates the cow's DMI every week depending on its fat corrected milk (FCM) production, live weight (LW) and DIM following equation 2 [2].

$$DMI_{dim,FCM,BW} = (0.372 * FCM_{dim} + 0.0968 * \mathrm{BW}^{0.75}) * \left(1 - \mathrm{e}^{-0.192*\left(\left(\frac{dim}{7}\right)+3.67\right)}\right) \qquad [2]$$

Where $DMI_{dim,FCM,LW}$ stands for dry matter intake (kg/cow/day) for a cow that weights BW at dim days in lactation and producing FCM kg.

At dry off, DMI was fixed to $DMI_{dry-off}$ (Table S1). From DMI's calculation, the UFL and MAT requirements are determined each week according cow's daily milk yield (DMY) ranging from 0,8 to 1 UFL per kg of DMI and from 0.10 to 0.17 MAT per kg of DMI as illustrated in Table S2.

1.3. Milk yield

Milk, milk fat and milk protein productions are simulated based on Wood's [3] lactation curve equation 3:

$$Y(wim) = 7 * \mathrm{a} * t^{b} * e^{-ct} \qquad [3]$$

Where $Y(wim)$ stands for weekly milk or fat or protein yield; a, b and c represent a scaling factor associated with the initial milk yield, the inclining slope up to peak yield, and the declining slope after peak yield, respectively.

Milk, fat and protein yields follows the standard lactation curve (b>0, c<0) (Silvestre et al., 2009). Each cow was randomly assigned a set of fixed constants a, b and c for her whole life according a normal distribution law. Milking duration is determined via the cow's reproductive performances, the dry period starting DRYLength weeks before the theoretical calving date, calculated to be PREGLength weeks after successful artificial insemination (AI) (Table S1)

1.4. Reproduction

Cow's reproduction simulation was based on a transition state machine between reproduction states (Figure S1). The transition from one status to another (cyclicity, heat expression, heat detection, insemination, pregnancy and calving) is conditioned by animal characteristics such as i) age and

bodyweight conditions for heifer's puberty and heifer eligibility to first AI (HeifAgePub, HeifWeiPub, HeifAgeElig, HeifWeiElig, see Table S3) and ii) heat expression (Pheat), heat detection (Pdetect) and successful insemination probabilities (Pinsem) for mature cows to start a pregnancy period (Table S3). During pregnancy period, abortion probabilities are applied every week (Pabort). New born gender is determined according to a GenderRatio. After calving, cow's ovarian cycle could start again after a 15-day anoestrus period and a 28-day voluntary waiting period (VWP), depending on cycle probability (Pcycle) and atypical cycles occurrence. Three main atypical cycles are considered: delayed cycle, prolonged luteal phase cycle and interrupted cycle.

1.5. Calves

Calves raising is also modelled on the week basis, as for cows. The feed requirement for calves and heifer were also adjusted on the weekly basis.

2- Diseases (production damage)

The health disorders were defined for each cow and each week mechanistically, based on basic incidences, cow specific risk factors and herd level risks (within herd contamination and farmer's practices). Diseases were implemented through alteration of the 3 production functions. The cow's diseases and treatments simulation included dystocia, subclinical hypocalcaemia, milk fever, placental retention, puerperal metritis, purulent vaginal discharge, subclinical endometritis, left and right abomasum displacement, lame, subclinical ketosis, clinical ketosis and mastitis.

Udder health contamination by six pathogens was considered, with a weekly risk occurrence (Staphylococcus aureus, Streptococcus spp., Klebsiella spp., Escherichia coli, negative cultures and others). Clinical mastitis infections occur during lactation (one pathogen maximum a week) depending on cow's lactation number, DIM, case number (first case, first relapse, second relapse or more), cow's diseases historic (milk fever, metritis, subclinical ketosis). During lactation, weekly somatic cells production is simulated through a basic SCC production as formulated in equations 4 and 5 [4] and an SCC increase in case of clinical mastitis infections presented in Table S3 [5].

$$SCC\_L1=1000\ (335\ EXP(-0{,}55\ wk) + 65 + 1\ wk) \qquad [4]$$

$$SCC\_Ls1=1000\ (335\ EXP(-0{,}55wk) + 65 + 1{,}5\ wk) \qquad [5]$$

Foot and claw diseases are simulated through a 'lame' function defining probabilistic weekly lame events and computes a 5 points lame score (LS) every week [6]. The score gains a point for every simulated lame event and loses 25% at cow's dry off. LS defines a cow's lame status and conditions its treatment.

Calves' health issues considered were stillbirth, neonatal diarrhoea, septicaemia, omphalitis and respiratory diseases up to 6 months old. Failure of passive immune transfer (colostrum intake) was considered to influence calf diseases risks. All the calf mortality was explained by these issues. Accidental issues (broken leg…) were excluded. Incidence risks parameters used to simulate calves' diseases are presented in Table S5.

For every simulated cow, the weekly occurrence of each disease depends on a computed final risk that accumulates (i) a basic incidence risk adjusted by cow characteristics (WIM, parity, milk yield) and following a disease calendar defining cow's susceptibility of diseased for each WIM or age (Tables S5 and S6), (ii) cross diseases risks (how one disease is influenced by another one) (Table S8) as relative risks applied on the basic incidence risks, (iii) management risks (farmers' routines and practices considered as scenario), (iv) and treatment related risk as explained in section 3.

3- Treatments (production damage control)

The damage control functions simulate treatments for sick cow and their consequences on health evolution for the treatment cows or herd. Dystocia, subclinical ketosis and failure immune transfer were yet excluded since no treatment was done in such a situation (due to subclinical status of the disease or management by intervention only). Probabilities of farmer or veterinarian interventions were determined by participatory approach and authors' experience (Table S6). Each treatment pattern is characterised by 3 items, namely (i) the treatment composition including drug (i.e. antimicrobials, anti-inflammatory...) and the nature of the intervention (cow-side intervention, consultation, surgery …) (ii) the expected efficiency on disease cure and expected relapse risk (i.e., non-treated clinical mastitis will increase the infection regular effects and the relapse risk by 50%) and (iii) three socio economic implications that are farmer's labour for disease management, treatment cost and veterinarian costs. Information about drugs application and composition were extracted from the official veterinary drugs index (Table S7).

For mastitis, treatment pattern is determined depending on (i) last month cow's SCC value (ii) the mastitis infection order during lactation (first, second etc) and the situation of recurrence or relapse (re infection after 3 weeks), (iii) and if the treatment is administrated on time, delayed or if mastitis not detected.

4- Herd dynamics and cows exit

A herd size objective was fixed for in-milk cows so as to consider barn constraint and a real in-milk cows herd-size was calculated weekly including new calvings (entry) and death (exit) to define cows to be culled (if needed). Cow death is accidental or consecutive to disease previously described. No

animal was bought and only calves raised in the farm were used as cows. Farmers keep only female calves. Male calves are sold at one-month age. Heifers ready to calve can be also sold two weeks before calving when the dairy capacity is full. No constraint on calves and heifer housing were considered.

To mimic the usual famer behaviour on cow culling for slaughterhouse, a set of rules was defined so as to make sure the culling decision depends on herd-size, herd production characteristics and health performances. Culling rules were applied on all cows each week, based on cow's milk yield, DIM, pregnancy status, lame score, and udder health status. These criteria represent the main criteria used by farmers for culling decision. The other health disorders were not considered in culling, but they act indirectly through production and reproduction performances and through udder health and lame.

Pre-culled animals represent cows according to their reproductive performances (failed inseminations and days open) that does not induce an immediate herd exit, but the abandonment of insemination attempts. The criteria and the thresholds used for culling decision change to stabilised the herd-size around the objective. Rules were relaxed or strengthened when herd-size change. Three culling politics were implemented depending on herd density. It starts at the first week with herd density reaching the considered threshold. At 80% herd density or less cows are only evaluated for culling through milk yield before 6 months' pregnancy (denoted P1). Between 95% and 110% density, cows are evaluated during 4 weeks with P1 rules and then with P2 rules (lame, udder health, milk quality production) during 4 weeks if density doesn't get reduced, and so on. If density is superior to 110%, P1 and P2 rules are applied all long, and an additional rule is applied through a combined score for SCC, lame and clinical mastitis. Details are reported in Table S8.

5- Economic optimization framework

The recursive mean–variance optimization framework economic model dynamically represented farmers' input allocation decisions while maximizing utility under constraints

The Markowitz–Freund mean–variance objective function was used to incorporate risk-averse behavior into farmers' decision-making [13–15]. The decision-maker's expected utility (F) can be represented as defined in Equation 6

$$\text{Max } F = E[Z_{k,t}] - 0.5\,\phi\sigma(Z_{k,t}) \qquad \textbf{[6]}$$

where F is the objective function of farmers, E denotes the expected values, k represents the state of nature (defined here as the possible price level), $Z_{k,t}$ is the income generated with state of nature k in year t, $\boldsymbol{\phi}$ is the risk aversion coefficient, and $\boldsymbol{\sigma}(Z_{k,t})$is the standard deviation of income. The risk

aversion coefficient was set to 1, and a sensitivity analysis was conducted for risk aversion coefficient values from 0 to 5, as these values represent different farmer attitudes toward risk. The income Z k,t generated with state of nature k in year t is equal to the difference between revenue and expenditures with state of nature k in year t.

Expenditures were calculated as the sum of health and veterinary expenses (e.g., purchases including antibiotics, veterinary consultations/interventions and surgery) (Ex_Vetk,t), feed expenses included expenditures due to strategy changes (e.g., purchases of concentrate) (Ex_Feedk,t) and other expenses (Ex_Othk,t) included related expense surcharges for housing and milking hygiene, insemination and other changed practices (Equation 7). Dairy revenues (Equation 8) were calculated as the sum of the revenues from each product sold, namely, milk (R_Milkk,t), one-month-old calves, heifers ready for calving (R_Anit) and cull meat (R_Cull)

$$Exk,t = Ex\_Vetk,t + Ex\_Feedk,t + Ex\_Othk,t \quad \textbf{[7]}$$

$$Rk,t = \sum LR\_Milkk,t + \sum AR\_Anit + R\_Cull \quad \textbf{[8]}$$

where L denotes the cytological qualities of milk and A denotes the type of animal sold (e.g., heifers or male calves).

The weekly milk quantities produced and sold by the farm were recorded, and the mean main weekly milk qualities (somatic cell count (SCC), fat and protein) determined the monthly milk price according to the usual payment criteria (Table S8).

Table S1. Biological model input parameters.

Parameters	Sources
Diseases risks and effects	[7–15]
Mastitis (clinical and subclinical) risks and effects	[4,5,9,10,12,16]
Lactation	[3,17,18]
Body weight and food needs	[1,2,19]
Heifers growth and reproduction	[9,14,20–24]
Reproduction parameters	[2,4,10,12,14,17,18,20–34]
Culling rules	[4,5,14,24,28,33,35,36]

Table S2. Production functions calibration parameters.

Label	Definition	Unit	Value	Reference
BW_{Birth}		kg	44	Authors
ADG		kg	< 6-month old : 0.65<br>up to first calving : 0.75	Authors
tpc		-	Pt - 50 > 0 : Pt - 50<br>Pt - 50 ≥ 0 : 0	[1]
k		-	0.0028	[37]
p1		kg	50	[1]
p2		days	75	[1]
p2		-	0.0187	[1]
BW_{MAT}		kg	U(650, 725)	Authors
$Carc_{Cull}$		%	55	Authors
$DMI_{dry-off}$		kg	13 ± 0.33	Authors
a (L1, L2, L3+)		-	7.418 ; 11.274 ;10.478	[17]
b (L1, L2, L3+)		-	0.429 ; 0.411 ; 0.447	[17]
c (L1, L2, L3+)		-	0.00525 ; 0.00666 ; 0.00725	[17]
DRYLength		weeks	7	Authors
PREGLength		weeks	40 ± 0.86	[38]
Pheat	Probability of heat	%	1st ovulation: 10<br>2sd ovulation: 50<br>> 3rd ovulation : 70	[31]
Pdetect		%	63	[23]
Pinsem		%	65	[23]
Pabort**	Probability of abortion, per month, for months 2 to 8	%	3.5; 2.5; 1.5; 0.5; 0.25; 0.1; 0.1	[27]
Pcycle***	Probability of cycling for WIM 1 to 8	%	PPa: 0; 10; 10; 10; 50; 50; 50; 100<br>MPa: 0; 10; 50; 50; 50; 100; 100; 100	Authors
Stillbirth		%	5	Authors
HeifAgePub		weeks	U(32, 58)	[23]
HeifWeiPub		kg	274	[23]
HeifAgeElig		weeks	59	[23]
HeifWeiElig		%BWMAT	U(55, 65)	[39]
GenderRatio		%	50%	Authors

[a] : PM : primiparous ; MP : multiparous

Table S3. UFL, crude protein and crude fiber food requirements calculation table.

Daily milk yields (kg/day)	Diet requirement		
	**UFL (/kg DMI)**	**Crude Proteins (% DMI*)**	**Crude Fiber (% DMI[a])**
0 (dry-off)	0,8-0,85	10 to 12	22-24
<20	0,85-0,90	13-14	20-22
20-25	0,90-0,93	14-15	19-21
25-30	0,93-0,97	15-16	18-20
30-35	0,95-1	16-16,5	17-19
>35	0,98-1	16,5-17	16-18

*: DMI: dry matter intake

References: https://www.quae.com/produit/1523/9782759228683/alimentation-des-ruminants

Table S4. Somatic cells count after clinical mastitis infection per pathogen.

.	Impact on SCC x 103				
	WkOcc1	WkOcc2	WkOcc3	WkOcc4	Daily slope after
Staphylococcus aureus	1644	344	231	118	-559
Streptococcus spp.	1774	374	264	154	-1100
Escherichia coli	1666	416	287	158	-559
Klebsiella spp.	1666	416	287	158	-559
Negative cultures	1440	493	363	233	-1317
Other cultures	1660	474	370	266	-999

Source: [4,5]

Table S5. Probability of treatment delay of clinical mastitis infections per pathogen.

	On time treatment			Delayed treatment			No treatment		
	1st Inf.	Recid.	Relap.	1st Inf.	Recid.	Relap.	1st Inf.	Recid.	Relap.
Staphylococcus aureus	50%	50%	100%	20%	20%	0%*	30%	30%	0%*
Streptococcus spp.	50%	50%	100%	20%	20%	0%*	30%	30%	0%*
Klebsiella spp.	90%	90%	0%	0%	0%	90%	10%	10%	10%
Escherichia coli	90%	90%	0%	0%	0%	90%	10%	10%	10%
Negative cultures	50%	50%	100%	20%	20%	0%*	30%	30%	0%*
Other cultures	50%	50%	100%	20%	20%	0%*	30%	30%	0%*

1st Inf : first clinical mastitis infection during laction; Recid. : Recidivism, new infection before 21days of the previous; Relap. : relapse, new infection 21 days after the previous infection
(*) All cows are treated on time if not culled

Table S6. Calibration parameters for calves' diseases simulation.

	Incidence			Intervention	
	**Weeks of age when applicable**	**Incidence risk for the period**	**Reference**	**Vet.**	**Farmer**
Failure immune tranfert	0 (birth)	N(0.1, 0.005)	[40]	0%	0%
Diarrhea	1 to 4	N(0.227, 0.127)	[40]	10%	90%
Bovine respiratory disease	1 to 26	N(0.114, 0.015)	[41]	10%	90%
Omphalitis	1 to 3	N(0.05, 0)	[40]	0%	100%
Septicemia	1 to 4	N(0.03, 0)	[40]	75%	25%

Table S7. Cows diseases calendar, cross diseases risk calibration and treatment intervention.

		Dystocia	Milk fever	Placental retention	Puerperal metritis	Purulent vaginal discharge	Subclinical endometritis	Displaced abomasum	Clinial and subclinial	Lame	Mastitis
**Week when applicable**											
Week in milk	0										
	1										
	2										
	3										
	4										
	5										
	6										
	7										
	8										
	>8										
**Cross diseases risk calibration map (columns on lines)**											
Dystocia											
Hypocalcaemia											
Placental retention											
Metritis											
Purulent vaginal discharge											
Subclinical endometritis											
Displaced abomasum											
Clinial and subclinial Ketosis											
Lame											
Mastitis											
**Treatment intervention**											
Veterinarian intervention		0%	50%	25%	85%	25%	0%	85%	50%	0%	0%
Farmer intervention		0%	50%	75%	15%	75%	100%	15%	50%	100%	100%

The red boxes represent the susceptibility of the cow during lactation according to the disease. the yellow boxes indicate that the occurrence of a disease (in column) induces a risk factor for the

occurrence of a disease in row. Treatment interventions indicates probabilities to define the treatment applicator.

Table S8. Characteristics of treatments used in DHS.

Commercial name	Molecule	Nature	Route	NA	Duration (days)	Milk WT (days)	Meat WT (days)
Duphamox_LA	Amoxicilline	AM	SC	1	3	2.5	21
Dexadreson	Dexamethasone	AIS	IM	1	1	3	8
Nuflor	Florfenicol	AM	SC	1	1	0	37
Draxxin100	Tulathromycin	AM	SC	1	1	0	22
Tolfine	Tolfenamic acid	AINS	IM	1	2	0	12
Shotapen	Benzylpenicillin	AM	IM	2	4	5	64
Potencil	Amoxicilline	AM	IM	2	3	0	10
Amphoprim	Sulfadimidine, trimethroprime	AM	IM	2	3	2	5
StopM	Penethamate	AM	IM	1	3	4	14
Colibolus	Colistine	AM	OR	1	3	0	5
Estrumate	Cloprostenol	H	IM	1	1	0	1
Bioveine_GMC	Acetylmethionine, choline, Glucose	ME	IV	2	1	0	0
Bioveine_Calcium	Calcium, Magnesium	ME	IV	0	1	0	0
Speciale2411	Sodium bicarbonate, Glucose, Saccharose	ME	IV	3	1	0	0
Rehydion	Sodium, Potassium, Glucose	REH	OR	3	2	0	0
Metricure	Cefapirin	AM	IVG	1	1	2	2
Lincocine	Lincomycin	AM	IMA	1	1.5	3.5	3
Mastijet	Neomycin	AM	IMA	3	2	4	30
Ubrolexin	Cefalexin	AM	IMA	2	2	5	10
Mastipeni	Benzylpenicillin	AM	IMA	2	2	6.5	7
Masticoli	Cloxacillin	AM	IMA	2	2	4	0
Synulox	Amoxicillin	AM	IMA	2	1.5	4	7
Clamoxyl_Oblets	Amoxicillin	AM	IU	1	1	0	1
Calform_Bolus	Magnesium	ME	OR	0	1		
Cepravin	Cefalonium	AM	IMA	1	1	6	21
Orbeseal	Teat sealant		IMA	0	1	0	0

Medecines are supposed to be used at their approved dose. NA denotes the number of applications per animal, MiWT milk withdrawal time and MeWT : meat withdrawal time. For treatments routes abbreviations, IM : intramuscular, IMA : intramammary, OR : oral, IU : intrauterine, IVG : intravaginal, IV : intravenous, SC : subcutaneous. For treatment nature abbreviations, AM : Antimicrobial, AIS : steroidal anti-inflammatory drug, AINS : Non-steroidal anti-inflammatory drug H : Hormones, ME : infusions for metabolic troubles, REH: rehydrating

Table S9. Culling decision-making calibration.

	Definition	Unit	Value
**Preculling rules calibration**			
DOCullL1	Days open to apply pre-culling option for first lactation cows	days	180
DOCullLs1	Days open to apply pre-culling option for second or more lactation cows	days	160
Numlact_precull	Cow parity threshold to be preculled (out of any other criteria)	-	6
NbAI_precull_Simp	Number of failed AI to be eligible for preculling	-	5
NbAI_precull_Comp	Number of AI to be eligible for preculling if milk production accouted for (DMYPrecull)	-	4
DMY_Precull	Daily milk yield threshold for preculling (when considered)	kg	25
**Culling rules calibration**			
DMY_Cull	threhsold may also be 3 or 5 % less producing cows the given week	kg	15
PregDur_Cull	Pregnancy duration threshold for culling	weeks	26
LameCow_Cull_Comp	Locomotion score to be eligible for culling if lame prevalence accounted for (LameHerd_Cull)	score	[3–4[
LameCow_Cull_Simp	Locomotion score to be eligible for culling	score	4
LameHerd_Cull	Percentage of cows with locomotion score > 3	%	15%
CMCow_Cull_Simp	Number of clinical mastitis during the current lactation to be eligible for culling	-	4
CMCow_Cull_Comp	Number of clinical mastitis during the current lactation to be eligible for culling if herd CM prevalence accounted for (CMHerdCull)	-	[2–4[
CMHerd_Cull	Percentage of cows with at least one CM last year	%	25
SCCCow_Cull_Simp	Average monthly SCC for the current lactation		900
SCCCowCull_Comp1	Last month average SCC for the current lactation if tank SCC accounted for (SCCBulkCullH) - High	103 cells /ml	250
SCCCowCull_Comp2	Last month average SCC for the current lactation if tank SCC accounted for (SCCBulkCullL) -Low		350
SCCBulk_CullH	Bulk milk SCC of the week	103	300
SCCBulk_CullL	Bulk milk SCC of the week	cells /ml	350

Table S10. Economic model calibration.

	Units	Values
Price_Vet1	€/intervention	20
Price_Vet2	€/intervention	35
Price_Vet3	€/intervention	45
Price_MilkP	€/kg	1.9
VarPrice_MilkP	-	10.53%
Price_CalvCcF	€/kg	0.3793
VarPrice_CalvCcF	-	14%
Price_Calfs	€/animal	80
VarPrice_Calfs	-	20%
Price_HeifersRTC	€/animal	1300
VarPrice_HeifersRTC	-	15.38%
Price_Soja	€/kg	0.2541
VarPrice_Soja	-	10.94%
Price_Cereal	€/kg	0.1834
VarPrice_Cereal	-	12.98%
Price_MeatC	€/kg carcass weight	2.5
NutrVal_Cereal_UFL	MFU per kg of food	1.03
NutrVal_Cereal_DMI	kg of dry matter per kg of food	0.862
NutrVal_Cereal_CP	-	10.8%
NutrVal_Soja_UFL	UFL per kg of food	1.08
NutrVal_Soja_DMI	kg of dry matter per kg of food	0.881
NutrVal_Soja_CP	-	35.4%
NutrVal_CornE_UFL	UFL per kg of food	0.35
NutrVal_CornE_DMI	kg of dry matter per kg of food	0.32
NutrVal_CornE_CP	-	2.87%
VarNutrVal_CornE_UFL	-	20%
VarNutrVal_CornE_DMI	-	20%
VarNutrVal_CornE_CP	-	20%
MilkPrice_Q1	€/kg of milk	[288.8 – 403.8]
Penalty_MilkQ2%Q1	€/kg of milk	3.1
Penalty_MilkQ3%Q1	€/kg of milk	9.2
Penalty_MilkQ4%Q1	€/kg of milk	15.3
Dev_MilkPrice	-	[2.3% - 7.7%]

Price_Vet denotes the price of veterinarian intervention by type (3 types were defined according to the treatment time); Price_MilkP: milk powder price; Price_CalvCcF: price of concentrated food for calves; Price_Calfs: 1-month-old male calf price; Price_HeifersRTC: price of heifers ready to calve; Price_Soja Soybean meal price; Price_Cereal: cereal-based concentrated food price; Price_MeatC: culled cow carcass weight price. Retrospective milk price analysis was performed to define the median price ranges (over 10 years) and their variations (Var parameters).

The Var parameters represent the coefficients of variation, computed as the ratio of the standard deviation to the mean for food and milk prices (cows and calves) and are defined based on experts for live animal prices and corn ensilage nutritional value variations. UFL: milk fodder unit, DMI: dry matter intake, and CP: crude protein

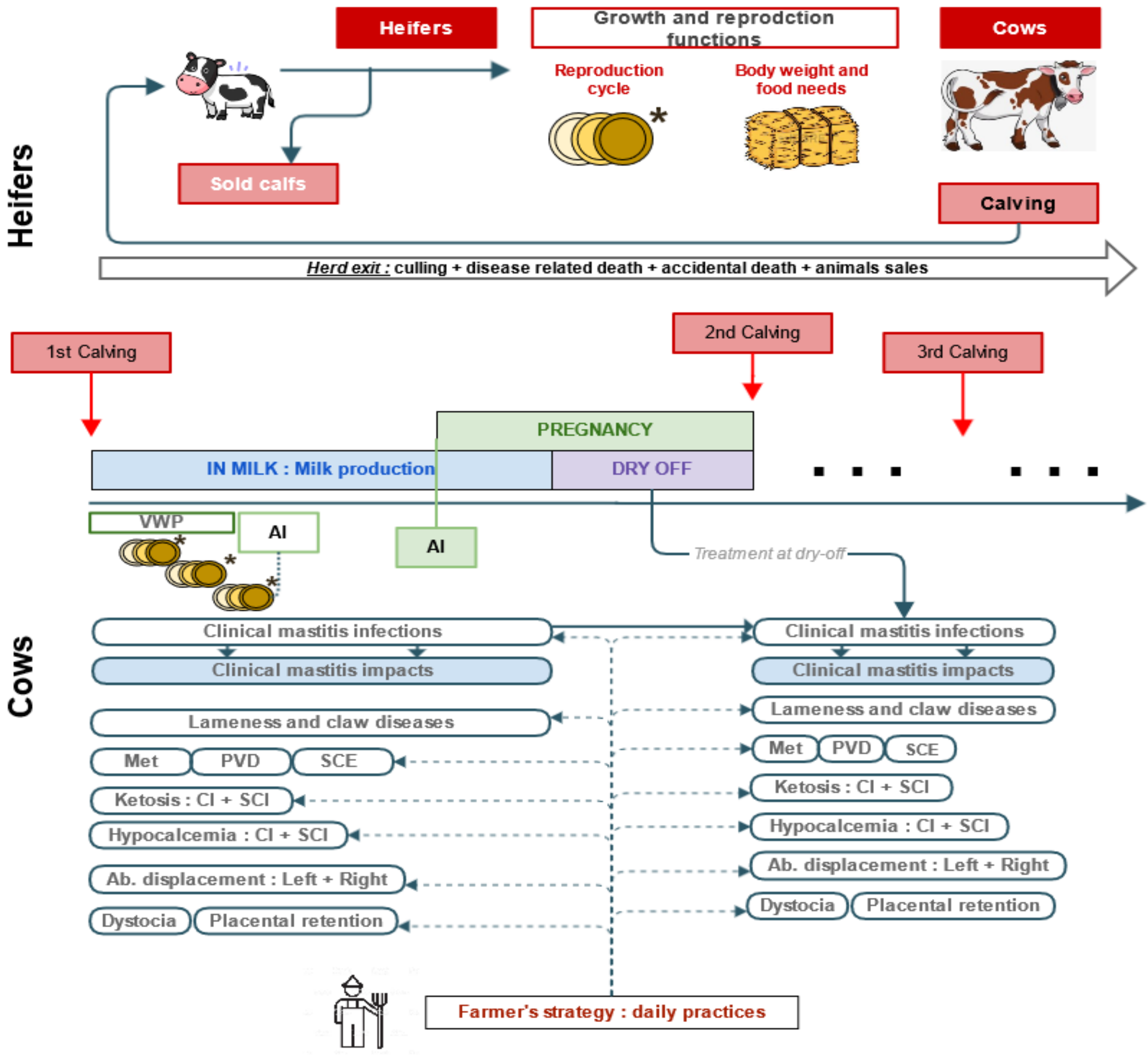

Figure S1. DHS biological model overview. VWP: farmer's voluntary waiting period before insemination, AI: artificial insemination, SCE: subclinical endometritis, PVD: purulent vaginal discharge, Met: metritis, Ketosis: clinical and subclinical ketosis, Hca: hypocalcemia and milk fever. (*) Cow reproduction was simulated as a state machine with atypical cycle simulation (Figure S1).

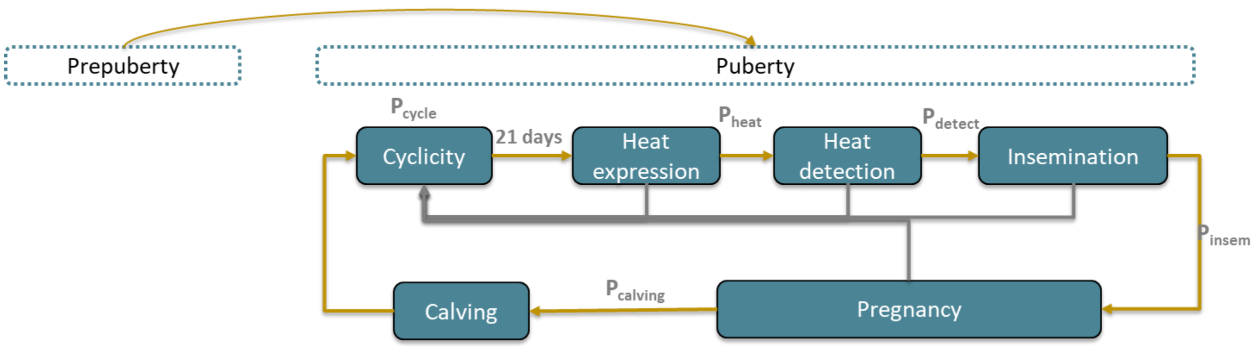

Figure S2. Reproduction simulation overview.

PART 2: ADDITIONNAL RESULTS

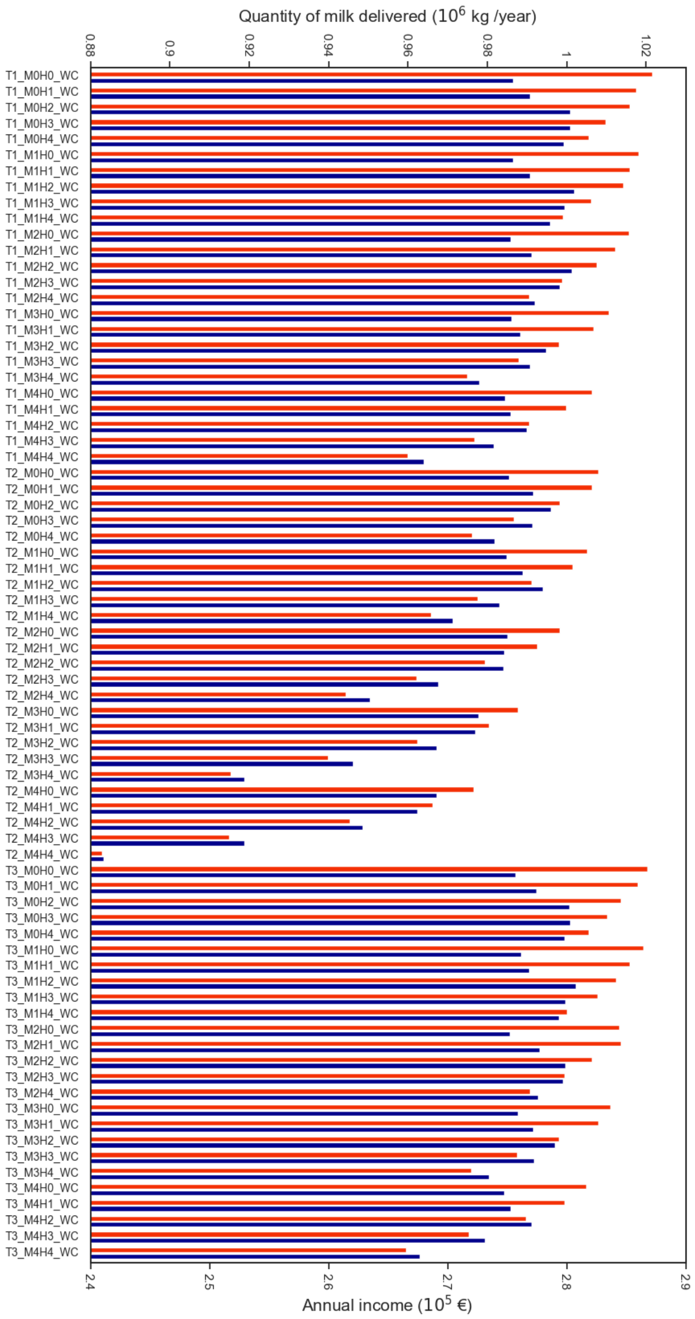

Figure S2.1. Quantity of milk delivered in tons (blue) and annual income in hundred thousand euros (red) for dry off treatment scenarios (T1, T2, T3), hygiene practices at milking parlor scenarios (M0, M1, M2, M3, M4) and hygiene practices at dairy housing scenarios (H0, H1, H2, H3, H4) for the strict cow threshold milk withdrawal strategy (WC).

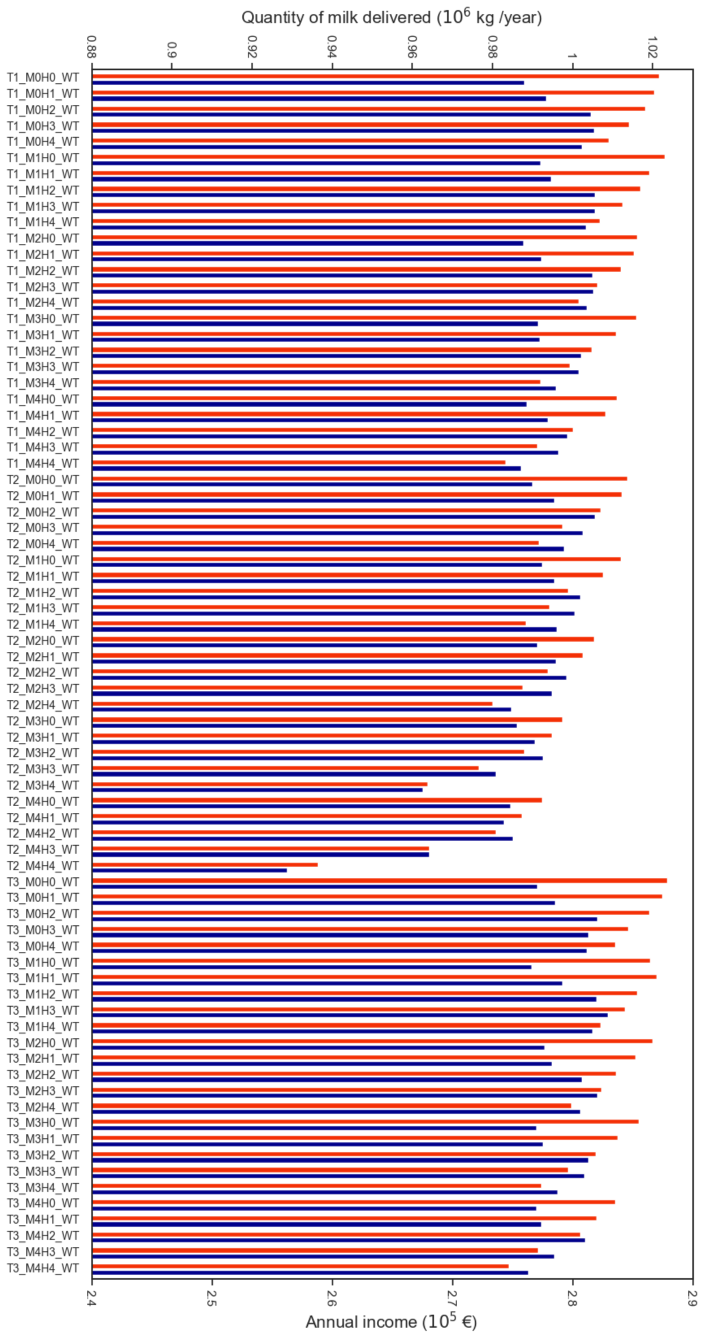

Figure S2.2. Quantity of milk delivered in tons (blue) and annual income in hundred thousand euros (red) for dry off treatment scenarios (T1, T2, T3), hygiene practices at milking parlor scenarios (M0, M1, M2, M3, M4) and hygiene practices at dairy housing scenarios (H0, H1, H2, H3, H4) for the mixed cow and tank threshold milk withdrawal strategy (WT)

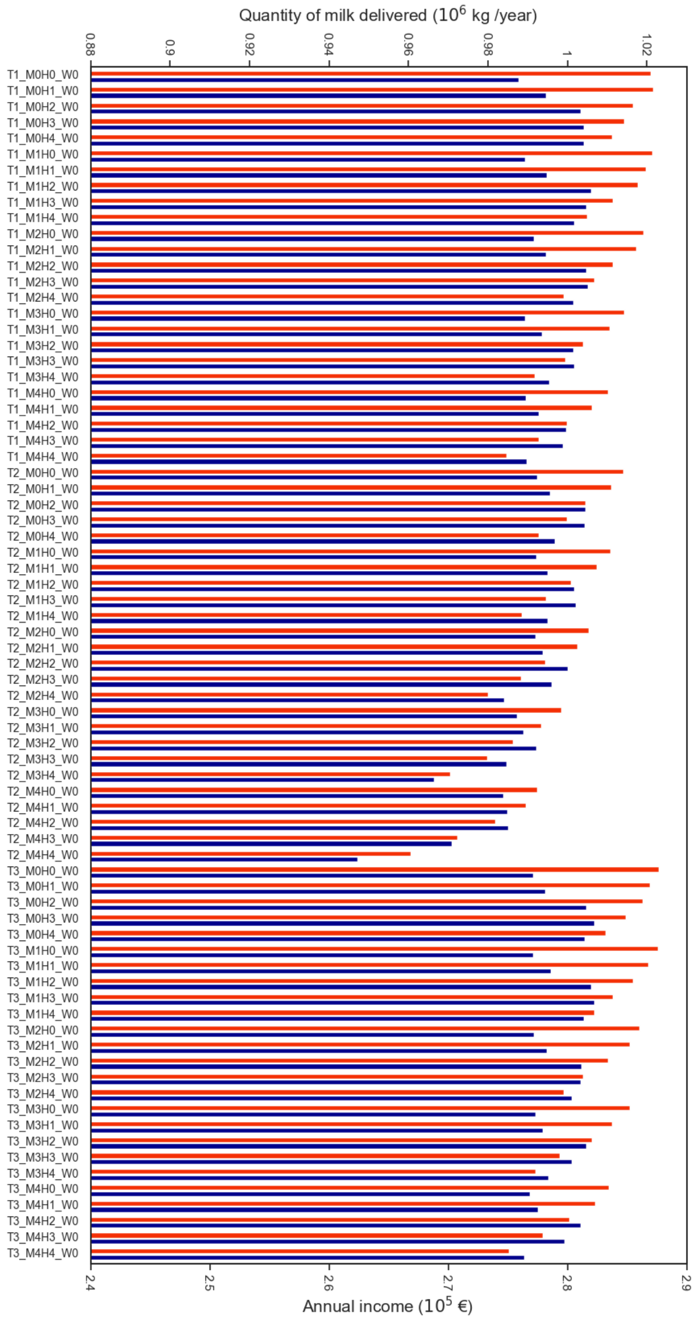

Figure S2.3. Quantity of milk delivered in tons (blue) and annual income in hundred thousand euros (red) under the dry-off treatment scenarios (T1, T2, and T3), milking parlor hygiene practice scenarios (M0, M1, M2, M3, and M4) and dairy housing hygiene practice scenarios (H0, H1, H2, H3, and H4) with the no-milk-withdrawal strategy (W0).

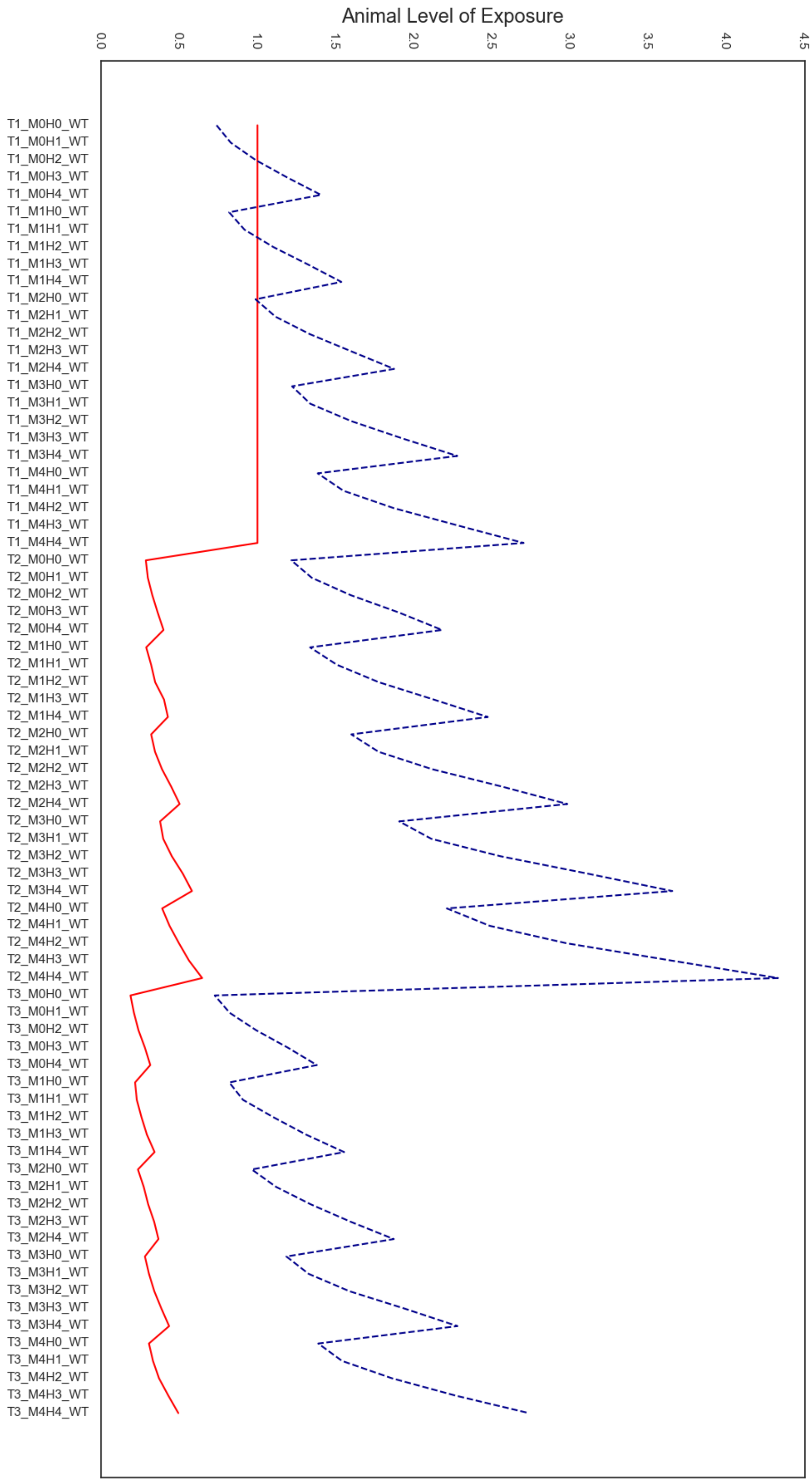

Figure S2.4. Animal Level of Exposure to Antimicrobials (ALEA) for all cows (blue color) and at dry-off (red color) for dry off treatment scenarios (T1, T2, T3), hygiene practices at milking parlor scenarios (M0, M1, M2, M3, M4) and hygiene practices at dairy housing scenarios (H0, H1, H2, H3, H4) for the mixed cow and tank threshold milk withdrawal strategy (WT).

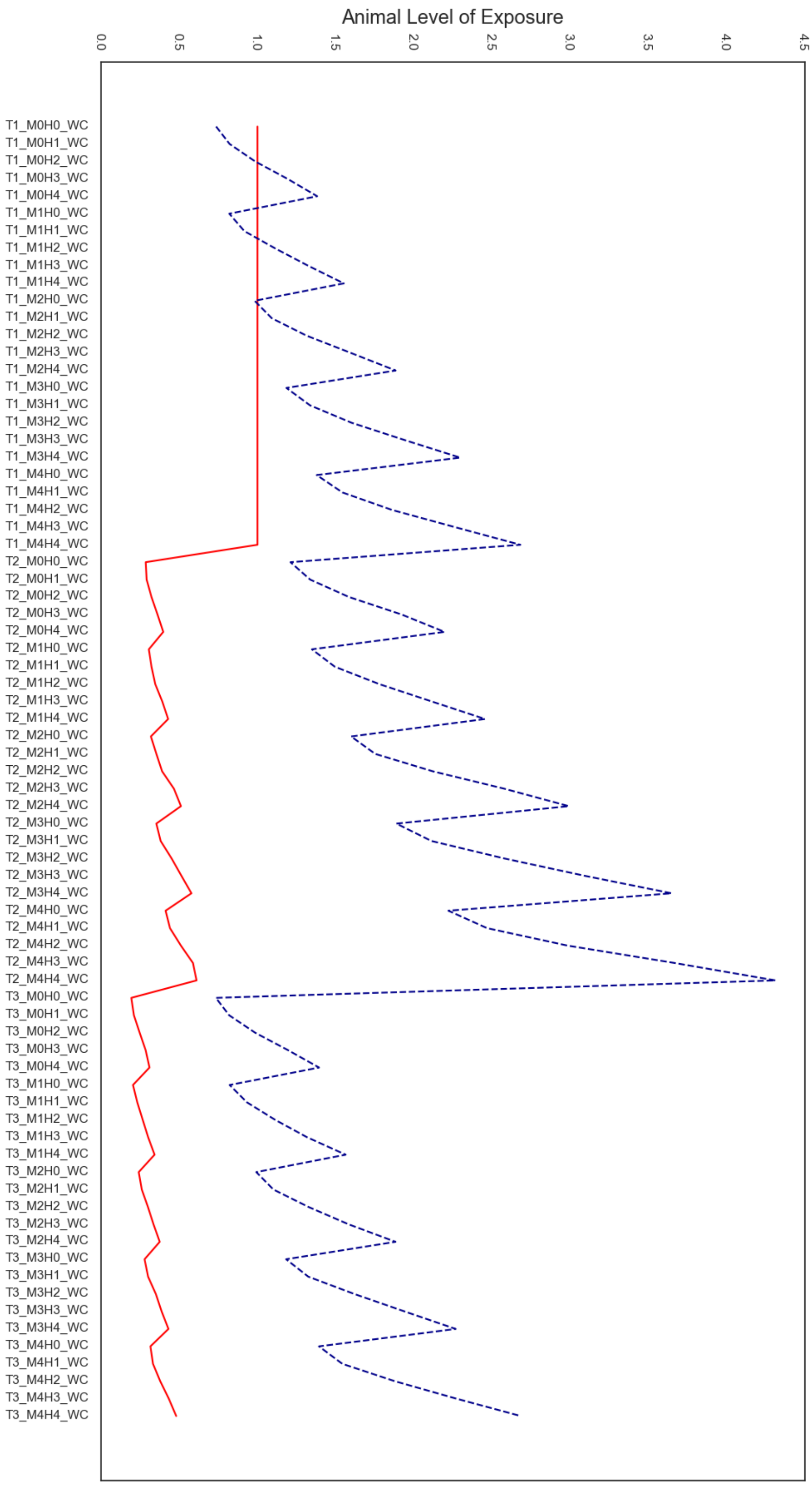

Figure S2.5. Animal Level of Exposure to Antimicrobials (ALEA) for all cows (blue color) and dry-off cows (red color) for dry off treatment scenarios (T1, T2, T3), hygiene practices at milking parlor scenarios (M0, M1, M2, M3, M4) and hygiene practices at dairy housing scenarios (H0, H1, H2, H3, H4) for the strict cow threshold (SCC) milk withdrawal strategy (WC)

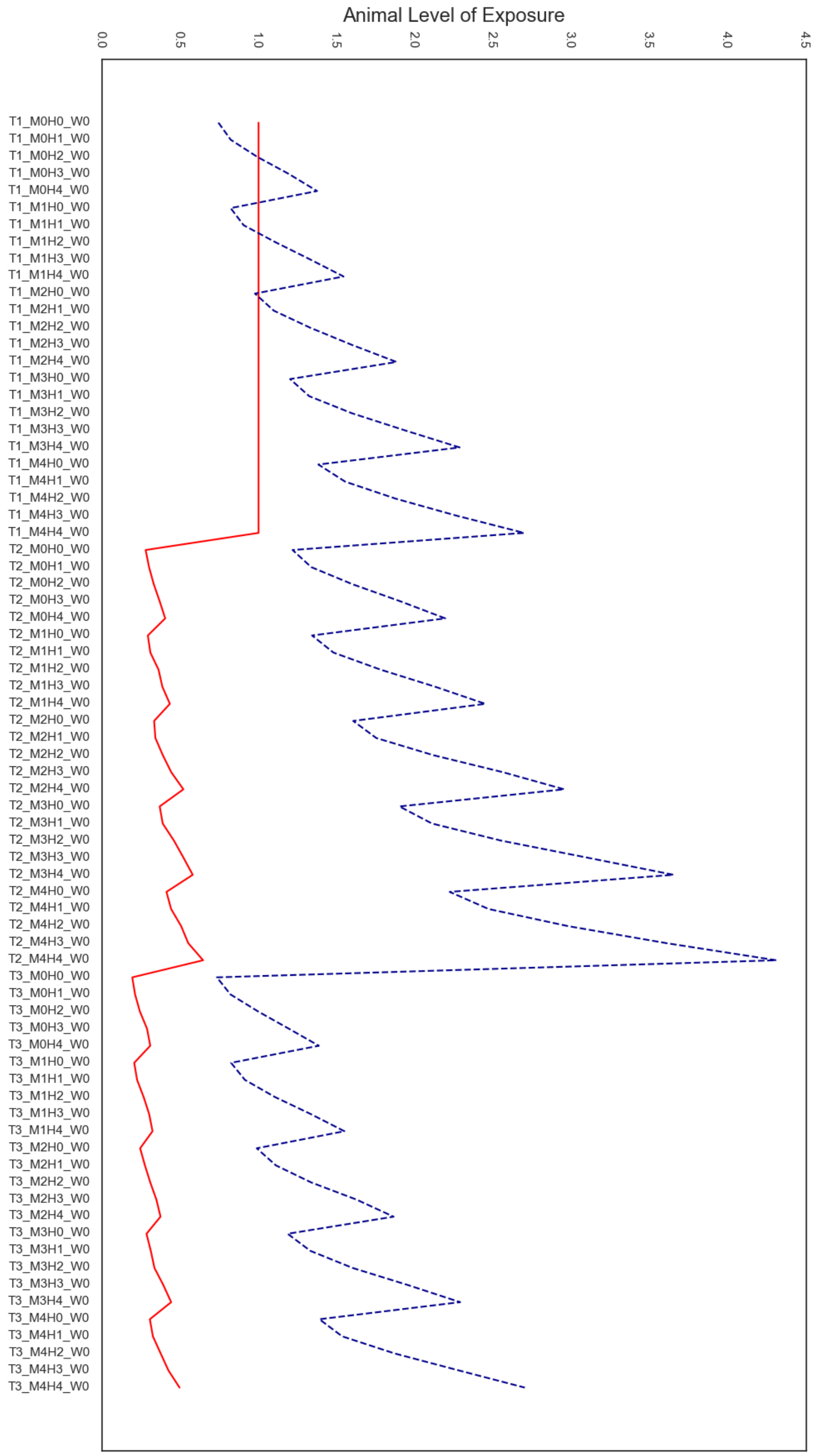

Figure S2.6. Animal level of exposure to antimicrobials (ALEA) for all cows (blue color) and at dry-off (red color) under the dry-off treatment scenarios (T1, T2, and T3), milking parlor hygiene practice scenarios (M0, M1, M2, M3, and M4) and dairy housing hygiene practice scenarios (H0, H1, H2, H3, and H4) with the no-milk-withdrawal strategy (W0).

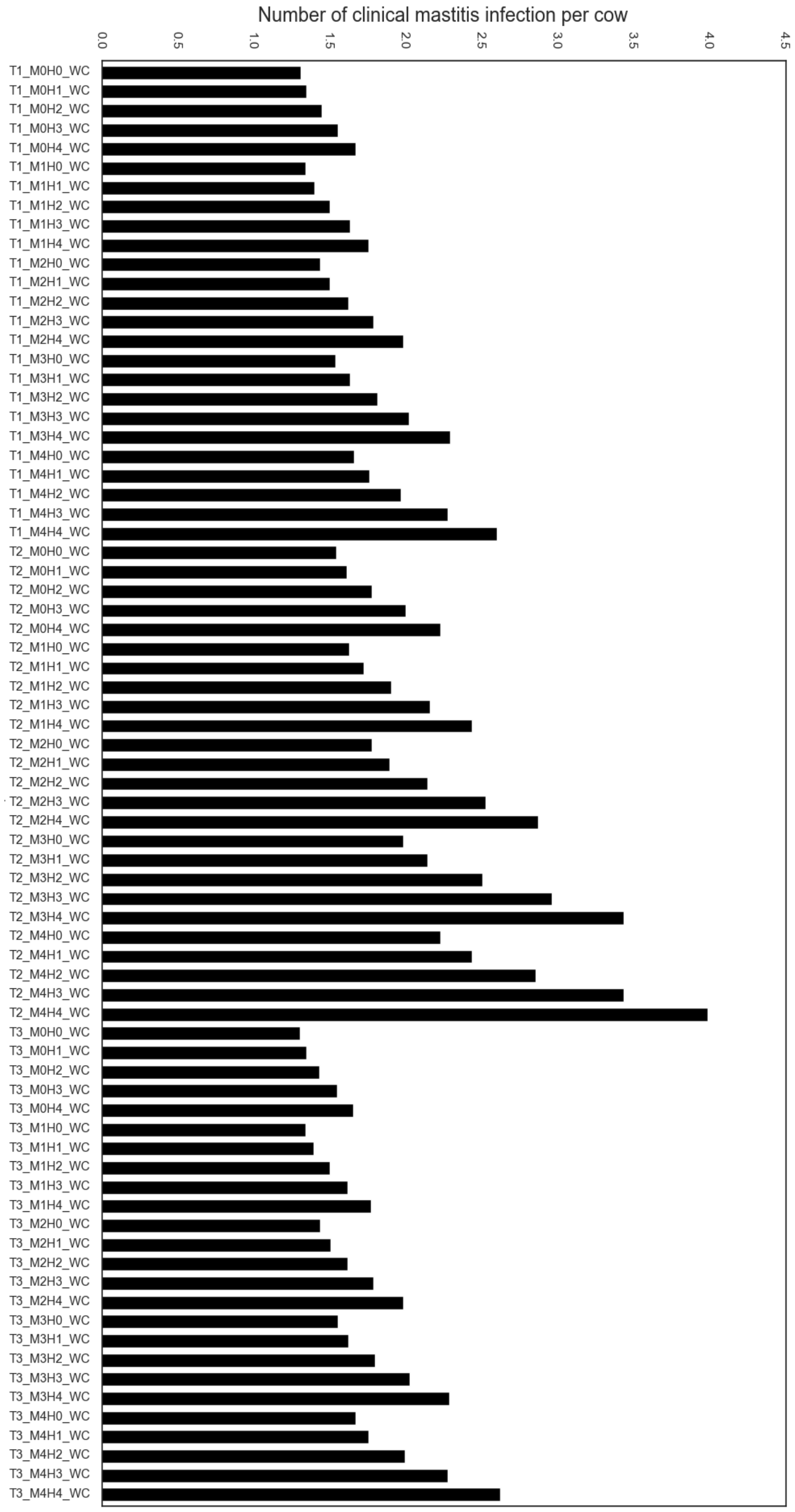

Figure S2.7. Number of clinical mastitis infection per cow per year for dry off treatment scenarios (T1, T2, T3), hygiene practices at milking parlor scenarios (M0, M1, M2, M3, M4) and hygiene practices at dairy housing scenarios (H0, H1, H2, H3, H4) for the strict cow threshold (SCC) milk withdrawal strategy (WC).

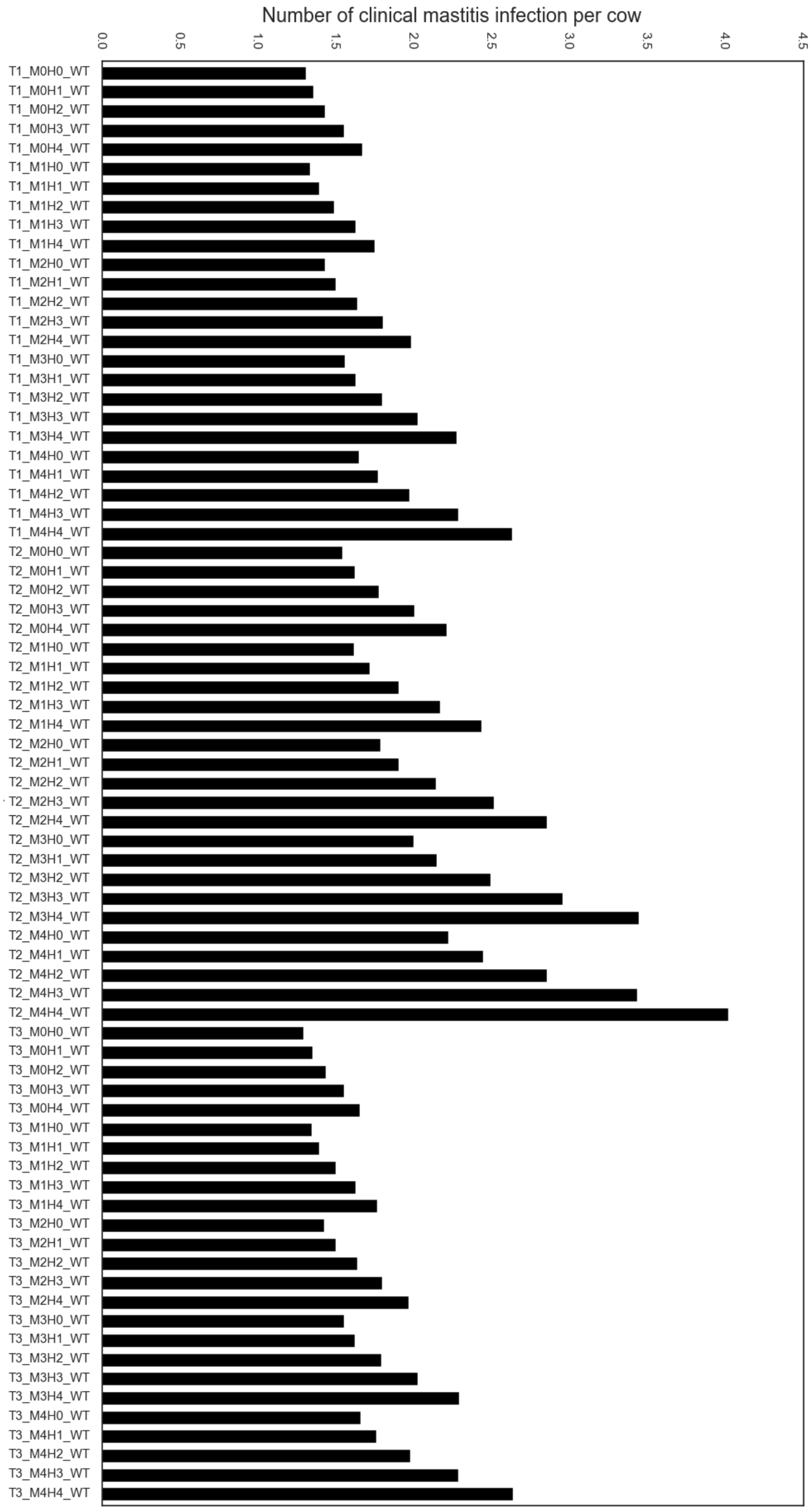

Figure S2.8. Number of clinical mastitis infection per cow per year for dry off treatment scenarios (T1, T2, T3), hygiene practices at milking parlor scenarios (M0, M1, M2, M3, M4) and hygiene practices at dairy housing scenarios (H0, H1, H2, H3, H4) for the mixed cow and tank threshold milk withdrawal strategy (WT).

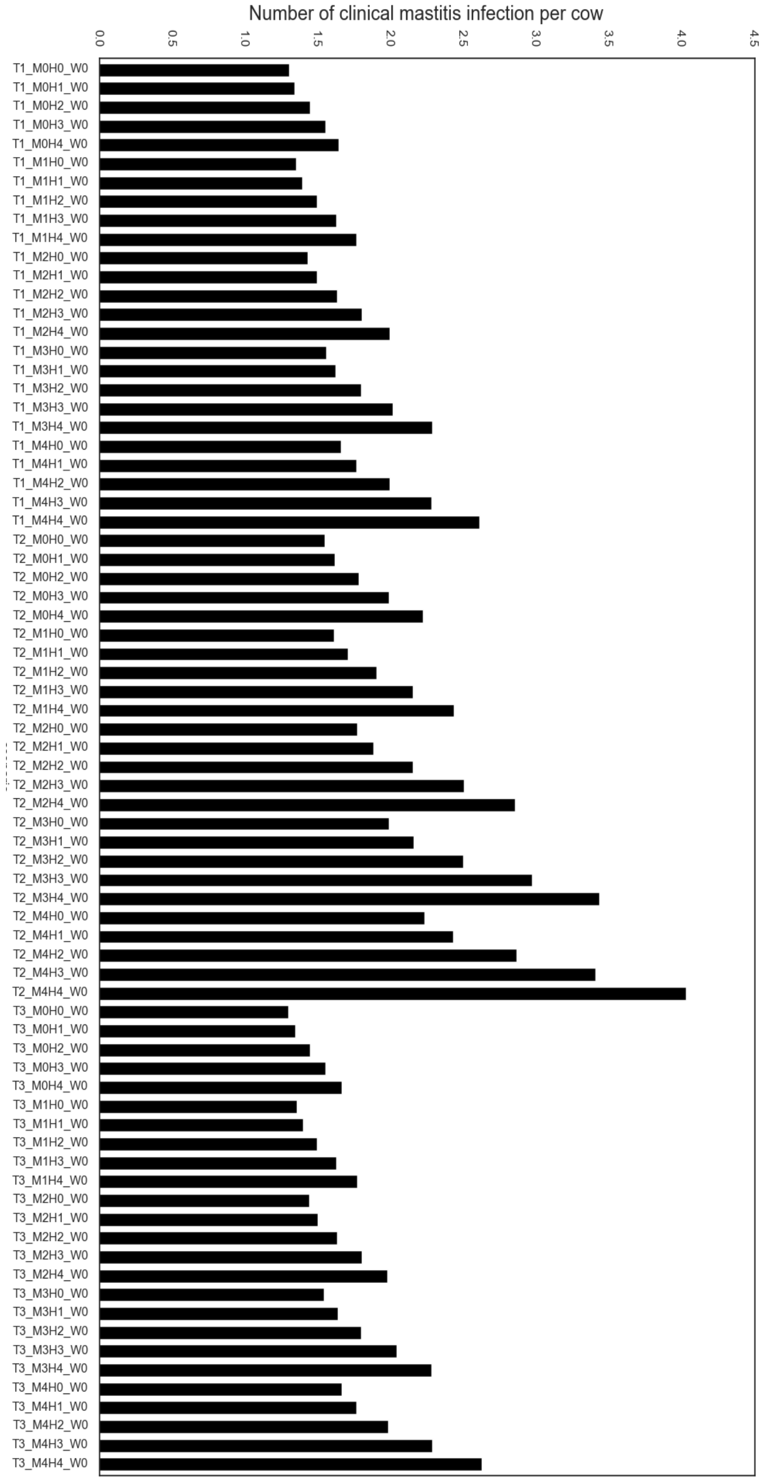

Figure S2.9. Number of clinical mastitis infections per cow under the dry-off treatment scenarios (T1, T2, and T3), milking parlor hygiene practice scenarios (M0, M1, M2, M3, and M4) and dairy housing hygiene practice scenarios (H0, H1, H2, H3, and H4) with the no-milk-withdrawal strategy (W0).

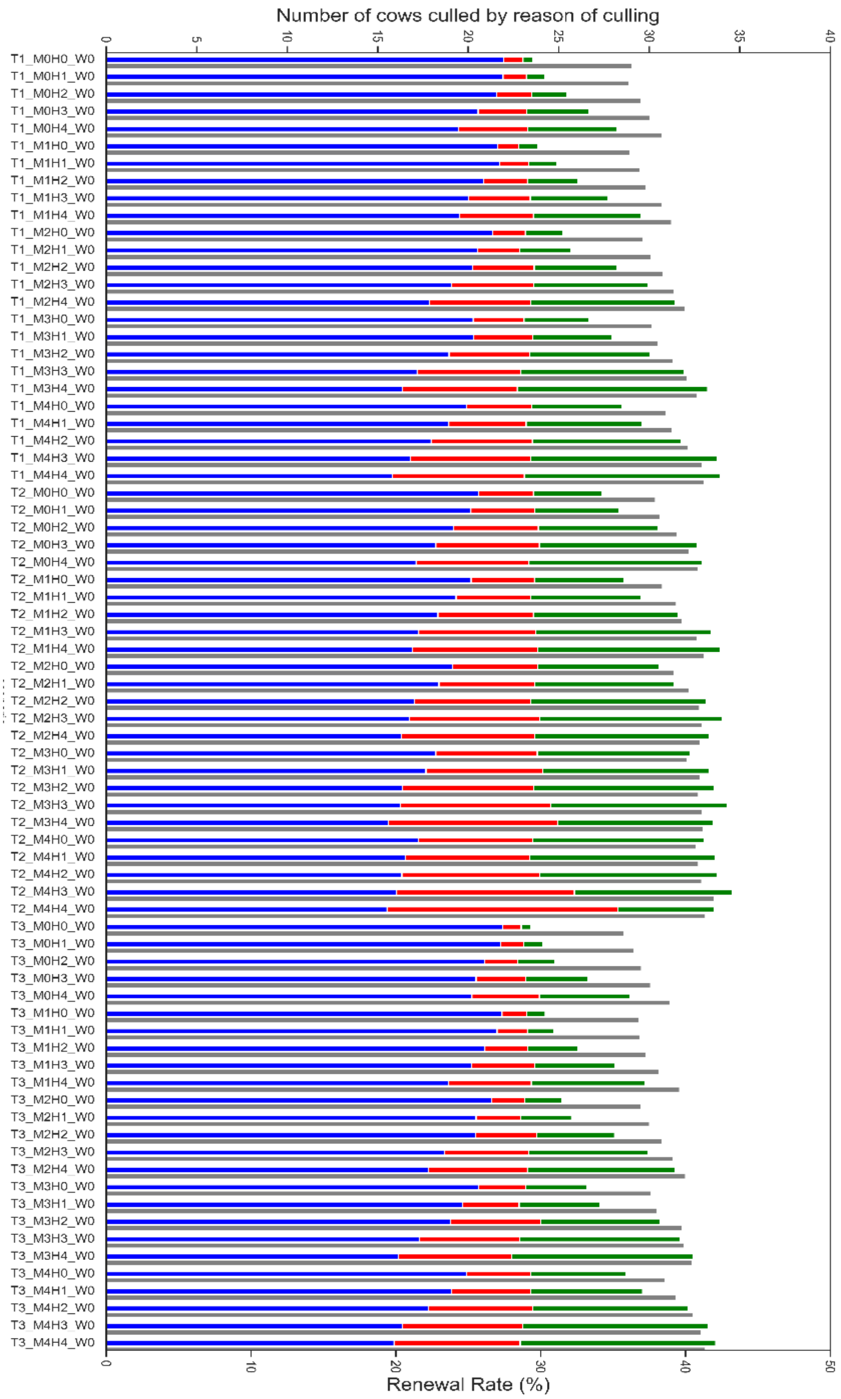

Figure S2.10. Renewal rate (Grey) and culling major reasons (colors) for dry off treatment scenarios (T1, T2, T3), hygiene practices at milking parlor scenarios (M0, M1, M2, M3, M4) and hygiene practices at dairy housing scenarios (H0, H1, H2, H3, H4) for the no milk withdrawal strategy (W0). Culling reasons represent cows culled because of dairy performance (blue); cows culled due to too high individual SCCs (red) and cows culled due to chronic or recurrent mastitis (green)

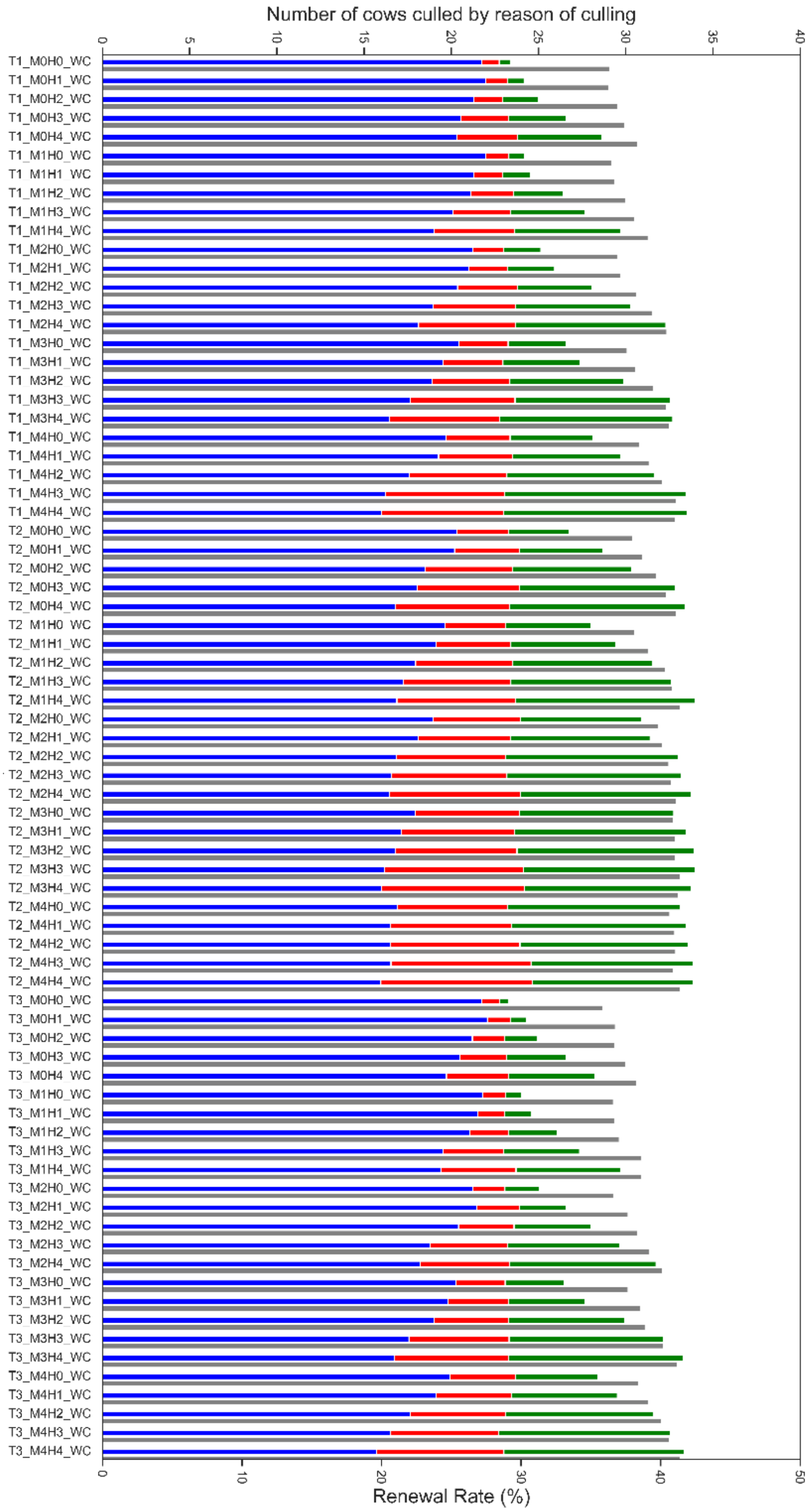

Figure S2.11. Renewal rate (Grey) and culling major reasons (colors) for dry off treatment scenarios (T1, T2, T3), hygiene practices at milking parlor scenarios (M0, M1, M2, M3, M4) and hygiene practices at dairy housing scenarios (H0, H1, H2, H3, H4) for the strict cow threshold (SCC) milk withdrawal strategy (WC) Culling reasons represent cows culled because of dairy performance (blue); cows culled due to too high individual SCCs (red) and cows culled due to chronic or recurrent mastitis (green)

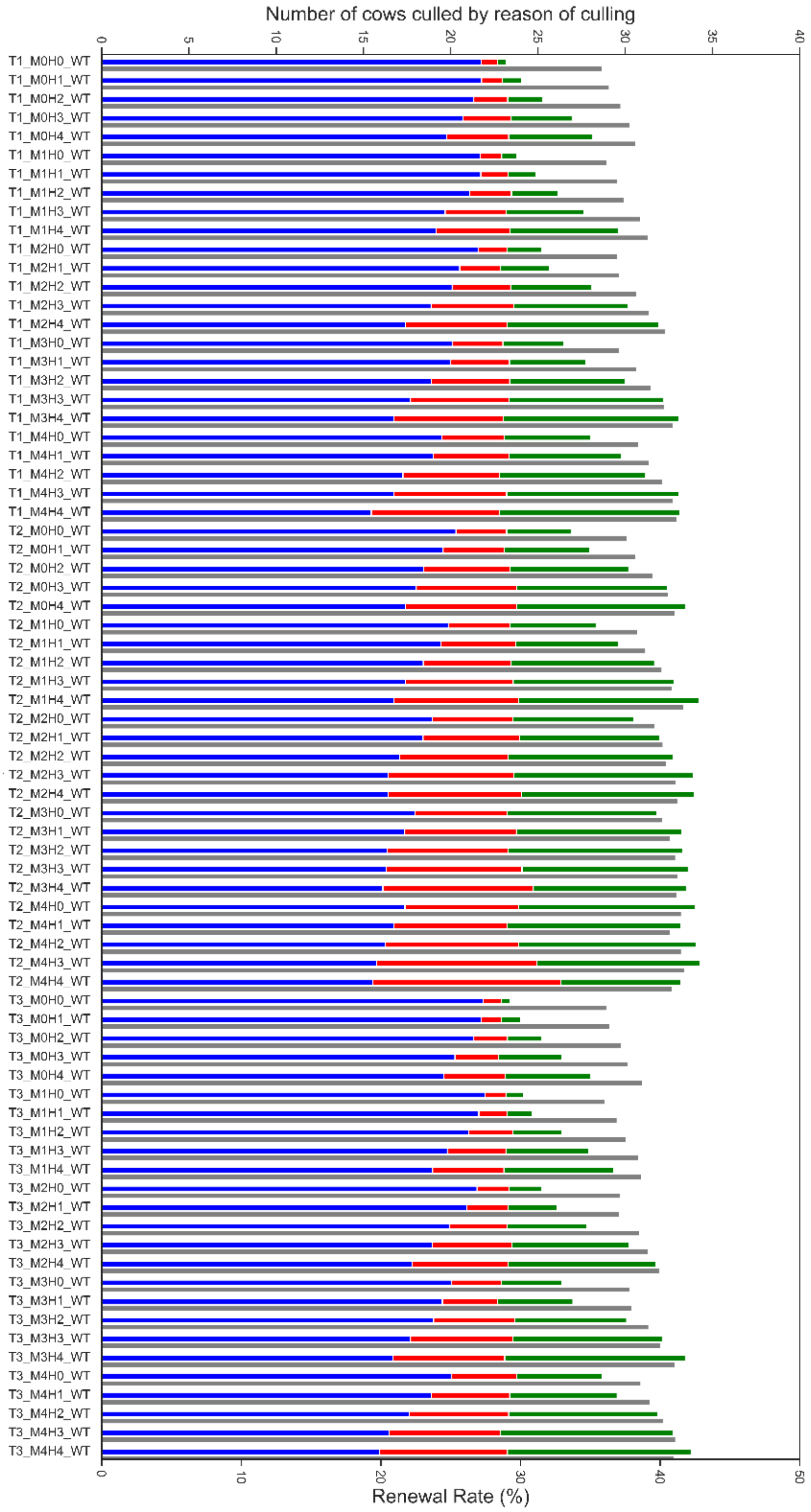

Figure S2.12. Renewal rate (Grey) and culling major reasons (colors) for dry off treatment scenarios (T1, T2, T3), hygiene practices at milking parlor scenarios (M0, M1, M2, M3, M4) and hygiene practices at dairy housing scenarios (H0, H1, H2, H3, H4) for the mixed cow and tank threshold (SCC) milk withdrawal strategy (WT). Culling reasons represent cows culled because of dairy performance (blue); cows culled due to too high individual SCCs (red) and cows culled due to chronic or recurrent mastitis (green)

References:

1. Van Arendonk, J.A.M. A Model to Estimate the Performance, Revenues and Costs of Dairy Cows under Different Production and Price Situations. Agric. Syst. 1985, 16, 157–189.

2. Giordano, J.O.; Kalantari, A.S.; Fricke, P.M.; Wiltbank, M.C.; Cabrera, V.E. A Daily Herd Markov-Chain Model to Study the Reproductive and Economic Impact of Reproductive Programs Combining Timed Artificial Insemination and Estrus Detection. J. Dairy Sci. 2012, 95, 5442–5460, doi:10.3168/jds.2011-4972.

3. Wood, P.D.P. Algebraic Model of the Lactation Curve in Cattle. Nature 1967, 216, 164–165.

4. Østergaard, S.; Chagunda, M.G.G.; Friggens, N.C.; Bennedsgaard, T.W.; Klaas, I.C. A Stochastic Model Simulating Pathogen-Specific Mastitis Control in a Dairy Herd. J. Dairy Sci. 2005, 88, 4243–4257, doi:10.3168/jds.S0022-0302(05)73111-8.

5. Cha, E.; Kristensen, A.R.; Hertl, J.A.; Schukken, Y.H.; Tauer, L.W.; Welcome, F.L.; Gröhn, Y.T. Optimal Insemination and Replacement Decisions to Minimize the Cost of Pathogen-Specific Clinical Mastitis in Dairy Cows. J. Dairy Sci. 2014, 97, 2101–2117, doi:10.3168/jds.2013-7067.

6. Sprecher, D.J.; Hostetler, D.E.; Kaneene, J.B. A Lameness Scoring System That Uses Posture and Gait to Predict Dairy Cattle Reproductive Performance. Theriogenology 1997, 47, 1179–1187, doi:10.1016/S0093-691X(97)00098-8.

7. Enting, H.; Kooij, D.; Dijkhuizen, A.A.; Huirne, R.B.M.; Noordhuizen-Stassen, E.N. Economic Losses Due to Clinical Lameness in Dairy Cattle. Livest. Prod. Sci. 1997, 49, 259–267, doi:10.1016/S0301-6226(97)00051-1.

8. Ostergaard, S.; Gröhn, Y.T. Effects of Diseases on Test Day Milk Yield and Body Weight of Dairy Cows from Danish Research Herds. J. Dairy Sci. 1999, 82, 1188–1201, doi:10.3168/jds.S0022-0302(99)75342-7.

9. Gröhn, Y.T.; Rajala-Schultz, P.J.; Allore, H.G.; DeLorenzo, M.A.; Hertl, J.A.; Galligan, D.T. Optimizing Replacement of Dairy Cows: Modeling the Effects of Diseases. Prev. Vet. Med. 2003, 61, 27–43, doi:10.1016/S0167-5877(03)00158-2.

10. Østergaard, S.; Sørensen, J.T.; Houe, H. A Stochastic Model Simulating Milk Fever in a Dairy Herd. Prev. Vet. Med. 2003, 58, 125–143, doi:10.1016/S0167-5877(03)00049-7.

11. Ettema, J.F.; Østergaard, S. Economic Decision Making on Prevention and Control of Clinical Lameness in Danish Dairy Herds. Livest. Sci. 2006, 102, 92–106, doi:10.1016/j.livprodsci.2005.11.021.

12. Raboisson, D.; Mounié, M.; Maigné, E. Diseases, Reproductive Performance, and Changes in Milk Production Associated with Subclinical Ketosis in Dairy Cows: A Meta-Analysis and Review. J. Dairy Sci. 2014, 97, 7547–7563, doi:10.3168/jds.2014-8237.

13. Raboisson, D.; Mounié, M.; Khenifar, E.; Maigné, E. The Economic Impact of Subclinical Ketosis at the Farm Level: Tackling the Challenge of over-Estimation Due to Multiple Interactions. Prev. Vet. Med. 2015, 122, 417–425, doi:10.1016/j.prevetmed.2015.07.010.

14. Mahnani, A.; Sadeghi-Sefidmazgi, A.; Cabrera, V.E. Consequences and Economics of Metritis in Iranian Holstein Dairy Farms. J. Dairy Sci. 2015, 98, 6048–6057, doi:10.3168/jds.2014-8862.

15. Raboisson, D.; Barbier, M. Economic Synergy between Dry Cow Diet Improvement and Monensin Bolus Use to Prevent Subclinical Ketosis: An Experimental Demonstration Based on Available Literature. Front. Vet. Sci. 2017, 4, 1–10, doi:10.3389/fvets.2017.00035.

16. de Haas, Y.; Barkema, H.W.; Veerkamp, R.F. The Effect of Pathogen-Specific Clinical Mastitis on the Lactation Curve for Somatic Cell Count. J. Dairy Sci. 2002, 85, 1314–1323, doi:10.3168/jds.S0022-0302(02)74196-9.

17. Meadows, C.; Rajala-Schultz, P.J.; Frazer, G.S. A Spreadsheet-Based Model Demonstrating the Nonuniform Economic Effects of Varying Reproductive Performance in Ohio Dairy Herds. J. Dairy Sci. 2005, 88, 1244–1254, doi:10.3168/jds.S0022-0302(05)72791-0.

18. Rutten, C.J.; Steeneveld, W.; Vernooij, J.C.M.; Huijps, K.; Nielen, M.; Hogeveen, H. A Prognostic Model to Predict the Success of Artificial Insemination in Dairy Cows Based on Readily Available Data. J. Dairy Sci. 2016, 99, 6764–6779, doi:10.3168/jds.2016-10935.

19. Friggens, N.C.; Ingvartsen, K.L.; Emmans, G.C. Prediction of Body Lipid Change in Pregnancy and Lactation. J. Dairy Sci. 2004, 87, 988–1000, doi:10.3168/jds.S0022-0302(04)73244-0.

20. Groenendaal, H.; Galligan, D.T.; Mulder, H.A. An Economic Spreadsheet Model to Determine Optimal Breeding and Replacement Decisions for Dairy Cattle. J. Dairy Sci. 2004, 87, 2146–2157, doi:10.3168/jds.S0022-0302(04)70034-X.

21. De Vries, A. Economic Value of Pregnancy in Dairy Cattle. J. Dairy Sci. 2006, 89, 3876–3885, doi:10.3168/jds.S0022-0302(06)72430-4.

22. Inchaisri, C.; Jorritsma, R.; Vos, P.L.A.M.; van der Weijden, G.C.; Hogeveen, H. Economic Consequences of Reproductive Performance in Dairy Cattle. Theriogenology 2010, 74, 835–846, doi:10.1016/j.theriogenology.2010.04.008.

23. Wathes, D.C.; Pollott, G.E.; Johnson, K.F.; Richardson, H.; Cooke, J.S. Heifer Fertility and Carry over Consequences for Life Time Production in Dairy and Beef Cattle. Animal 2014, 8, 91–104, doi:10.1017/S1751731114000755.

24. Mohd Nor, N.; Steeneveld, W.; Mourits, M.C.M.; Hogeveen, H. The Optimal Number of Heifer Calves to Be Reared as Dairy Replacements. J. Dairy Sci. 2015, 98, 861–871, doi:10.3168/jds.2014-8329.

25. Opsomer, G.; Coryn, M.; de Kruif, A. Measurement of Ovarian Cyclicity in the Post Partum Dairy Cow by Progesterone Analysis. Reprod. Domest. Anim. 1999, 34, 297–300, doi:10.1111/j.1439-0531.1999.tb01255.x.

26. de Vries, A. Economics of Delayed Replacement When Cow Performance Is Seasonal. J. Dairy Sci. 2004, 87, 2947–2958, doi:10.3168/jds.S0022-0302(04)73426-8.

27. Santos, J.E.P.; Juchem, S.O.; Cerri, R.L.A.; Galvão, K.N.; Chebel, R.C.; Thatcher, W.W.; Dei, C.S.; Bilby, C.R. Effect of BST and Reproductive Management on Reproductive Performance of Holstein Dairy Cows. J. Dairy Sci. 2004, 87, 868–881, doi:10.3168/jds.S0022-0302(04)73231-2.

28. Kristensen, E.; Østergaard, S.; Krogh, M.A.; Enevoldsen, C. Technical Indicators of Financial Performance in the Dairy Herd. J. Dairy Sci. 2008, 91, 620–631, doi:10.3168/jds.2007-0201.

29. Cabrera, V.E. A Large Markovian Linear Program to Optimize Replacement Policies and Dairy Herd Net Income for Diets and Nitrogen Excretion. J. Dairy Sci. 2010, 93, 394–406, doi:10.3168/jds.2009-2352.

30. Ettema, J.; Østergaard, S.; Kristensen, A.R. Modelling the Economic Impact of Three Lameness Causing Diseases Using Herd and Cow Level Evidence. Prev. Vet. Med. 2010, 95, 64–73, doi:10.1016/j.prevetmed.2010.03.001.

31. Inchaisri, C.; Jorritsma, R.; Vos, P.L.A.M.; van der Weijden, G.C.; Hogeveen, H. Analysis of the Economically Optimal Voluntary Waiting Period for First Insemination. J. Dairy Sci. 2011, 94, 3811–3823, doi:10.3168/jds.2010-3790.

32. Kalantari, A.S.; Cabrera, V.E. The Effect of Reproductive Performance on the Dairy Cattle Herd Value Assessed by Integrating a Daily Dynamic Programming Model with a Daily Markov Chain Model. J. Dairy Sci. 2012, 95, 6160–6170, doi:10.3168/jds.2012-5587.

33. Rutten, C.J.; Steeneveld, W.; Inchaisri, C.; Hogeveen, H. An Ex Ante Analysis on the Use of Activity Meters for Automated Estrus Detection: To Invest or Not to Invest? J. Dairy Sci. 2014, 97, 6869–6887, doi:10.3168/jds.2014-7948.

34. Shahinfar, S.; Guenther, J.N.; David Page, C.; Kalantari, A.S.; Cabrera, V.E.; Fricke, P.M.; Weigel, K.A. Optimization of Reproductive Management Programs Using Lift Chart Analysis and Cost-Sensitive Evaluation of Classification Errors. J. Dairy Sci. 2015, 98, 3717–3728, doi:10.3168/jds.2014-8255.

35. Sørensen, J.T.; Kristensen, E.S.; Thysen, I. A Stochastic Model Simulating the Dairy Herd on a PC. Agric. Syst. 1992, 39, 177–200, doi:10.1016/0308-521X(92)90107-Y.

36. Dechow, C.D.; Goodling, R.C. Mortality, Culling by Sixty Days in Milk, and Production Profiles in High- and Low-Survival Pennsylvania Herds. J. Dairy Sci. 2008, 91, 4630–4639, doi:10.3168/jds.2008-1337.

37. Korver, S. Efficiency of Breeds for Milk and Beef Production 1984.

38. De Vries, A. Improved Accuracy of a Model to Optimize Breeding and Replacement Decisions for Dairy Cattle. In Proceedings of the Computers in Agriculture and Natural Resources, 23-25 July 2006, Orlando Florida; American Society of Agricultural and Biological Engineers, 2006; p. 624.

39. Margerison, J.; Downey, N. Guidelines for Optimal Dairy Heifer Rearing and Herd Performance. Calf Heifer Rearing Princ. Rearing Mod. Dairy Heifer Calf Calving Nottm. Univ. Press Nottm. 2005, 307–338.

40. Raboisson, D.; Trillat, P.; Cahuzac, C. Failure of Passive Immune Transfer in Calves: A Meta-Analysis on the Consequences and Assessment of the Economic Impact. PloS One 2016, 11, e0150452–e0150452, doi:10.1371/journal.pone.0150452.

Rio, O. Fréquences et risques de mortalité et troubles de santé des veaux en élevage laitier. Thèse Méd. Vét. (École nationale vétérinaire de Nantes: Nantes, 1999).
